# Visceral Leishmaniasis in the Indian Subcontinent: Modelling Epidemiology and Control

**DOI:** 10.1371/journal.pntd.0001405

**Published:** 2011-11-29

**Authors:** Anette Stauch, Ram Rup Sarkar, Albert Picado, Bart Ostyn, Shyam Sundar, Suman Rijal, Marleen Boelaert, Jean-Claude Dujardin, Hans-Peter Duerr

**Affiliations:** 1 Department of Medical Biometry, University of Tübingen, Tübingen, Germany; 2 Centre for Cellular and Molecular Biology (CSIR), Hyderabad, India; 3 Institute of Tropical Medicine, Antwerp, Belgium; 4 Institute of Medical Sciences, Banaras Hindu University, Varanasi, India; 5 Koirala Institute of Medical Sciences, Dharan, Nepal; 6 Laboratory for Microbiology, Parasitology and Hygiene, Department of Biomedical Sciences, Antwerp University, Antwerp, Belgium; Yale University, United States of America

## Abstract

**Background:**

In the Indian subcontinent, about 200 million people are at risk of developing visceral leishmaniasis (VL). In 2005, the governments of India, Nepal and Bangladesh started the first regional VL elimination program with the aim to reduce the annual incidence to less than 1 per 10,000 by 2015. A mathematical model was developed to support this elimination program with basic quantifications of transmission, disease and intervention parameters. This model was used to predict the effects of different intervention strategies.

**Methods and Findings:**

Parameters on the natural history of *Leishmania* infection were estimated based on a literature review and expert opinion or drawn from a community intervention trial (the KALANET project). The transmission dynamic of *Leishmania donovani* is rather slow, mainly due to its long incubation period and the potentially long persistence of parasites in infected humans. Cellular immunity as measured by the Leishmanin skin test (LST) lasts on average for roughly one year, and re-infection occurs in intervals of about two years, with variation not specified. The model suggests that transmission of *L. donovani* is predominantly maintained by asymptomatically infected hosts. Only patients with symptomatic disease were eligible for treatment; thus, in contrast to vector control, the treatment of cases had almost no effect on the overall intensity of transmission.

**Conclusions:**

Treatment of Kala-azar is necessary on the level of the individual patient but may have little effect on transmission of parasites. In contrast, vector control or exposure prophylaxis has the potential to efficiently reduce transmission of parasites. Based on these findings, control of VL should pay more attention to vector-related interventions. Cases of PKDL may appear after years and may initiate a new outbreak of disease; interventions should therefore be long enough, combined with an active case detection and include effective treatment.

## Author Summary

Visceral Leishmaniasis is a neglected, life-threatening disease affecting the poorest of the poor. It has received more attention in light of the regional VL elimination program. A deterministic compartmental model was developed to estimate parameters for *L. donovani* transmission and to optimise intervention success. Our simulation results show that transmission of *L. donovani* is predominantly driven by asymptomatically infected hosts who are not eligible for treatment. Treatment can reduce the prevalence of symptomatic disease, but the incidence of KA remains on similar levels because of an unchanged intensity of transmission. In contrast to treatment-related interventions, vector-related interventions have the potential to reduce the prevalence of asymptomatic infections and thus are the intervention of choice from an epidemiological perspective. Vector control, however, should be combined with treatment, as PKDL cases can act as reservoirs of infection. This reservoir function originates from the long period of nearly two years on average during which putatively recovered KA patients develop PKDL.

## Introduction

Leishmaniasis is a vector-borne disease, which is caused by protozoan flagellates and is transmitted by phlebotomine sand flies. Clinical manifestations of leishmaniasis include cutaneous leishmaniasis (CL), muco-cutaneous leishmaniasis (MCL), visceral leishmaniasis (VL) and post-kala-azar dermal leishmaniasis (PKDL). The only life-threatening form is VL, which is strongly linked with poor housing [1]. The majority of VL cases (>90%) occur in only six countries: Bangladesh, India, Nepal, Sudan, Ethiopia and Brazil [2]. In East Africa and the Indian subcontinent, the disease is caused by *L. donovani*. In Europe, North Africa and Latin America *L. infantum* predominates [3]. In the Indian subcontinent, about 200 million people are estimated to be at risk of developing VL; this region harbours an estimated 67% of the global VL disease burden [2]. In this region, VL has been endemic for many decades, with the first reported epidemic in Bengal in the 1820s [4].

### Disease

VL, also called Kala-azar (KA), causes more than 50,000 deaths each year worldwide [5]. The name ‘Kala-azar’ (‘black-fever’) originates from India; it refers to the hyperpigmentation of the skin during the course of the disease. In contrast to CL and MCL, where the parasites are localised in tegumentary tissue, VL is a systemic infection of the phagocytic and reticulo-endothelial system; this infection includes the lymph nodes, spleen and liver. Clinical symptoms of VL are prolonged fever, fatigue, weight loss, bleeding tendency and an enlarged spleen and liver. Pancytopenia is also a characteristic sign.

PKDL, first described by Brahmachari in 1922 [6], can occur as a sequel to KA. It usually develops 6 months to several years after treatment and putative recovery from KA [7]. Poor treatment compliance is thought to favour the occurrence of PKDL [8], but a small fraction of PKDL cases have no reported history of KA [9]. The nodular lesions of PKDL patients usually contain many parasites [10]. PKDL is not a life-threatening condition, laboratory confirmation is challenging, and the treatment burdens the patients (in Bangladesh, current treatment guidelines call for 120 intramuscular injections of sodium stibogluconate [9]). Therefore, it is assumed that many patients remain undiagnosed and untreated, and PKDL patients are thought to act as a reservoir of infection [11].

Cases with VL and HIV co-infection have been reported from 35 countries. Both cellular and humoral responses to *Leishmania* are diminished in such patients [12], leading to an increased risk of developing VL after *Leishmania* infection and a higher rate of treatment failure [13], [14]. The rate of HIV-VL coinfection in the Indian subcontinent is still low but rising in Ethiopia. HIV-coinfected patients are relevant for the study of transmission, as they are more infectious due to higher parasitaemia [15].

### Diagnostics

The classical confirmatory test for VL used to be the microscopic detection of amastigote parasites from aspirates of lymph nodes, bone marrow or spleen [16]. This invasive method has largely been replaced by antibody-detection using rK39 immunochromatographic tests or by the direct agglutination test (DAT) [2]. Several PCR techniques have been developed. They seem to be more sensitive for detecting asymptomatic infections than the antibody tests [17], as it is hypothesised that PCR of peripheral blood will be the first marker to become positive after infection (before antibody seroconversion takes place). The leishmanin skin test (LST) is a marker of the delayed-type hypersensitivity reaction and is used in epidemiological surveys to study what proportion of the population has become ‘immune’ to leishmanial infection after exposure.

### Transmission cycle

When taking a bloodmeal on an infected host, female sand flies ingest the amastigote form of the parasites. In the sand fly gut, the parasites develop into procyclic promastigote flagellar forms, which divide and later on differentiate into metacyclic promastigotes. These forms migrate to the pharyngeal valve and can then be transmitted at subsequent blood meals. In the human host, parasites change back to amastigotes and multiply in cells of the mononuclear phagocyte system, impeding the immune defence mechanisms of macrophages [18].

### Control strategies

In 2005, the governments of India, Nepal and Bangladesh started the first regional VL elimination program, aiming to reduce the annual incidence of VL to less than 1 per 10,000 by 2015. The program focuses on treatment-related control strategies, such as early diagnosis and complete treatment, and vector-related control strategies, including indoor residual insecticide spraying.

### Treatment

Currently, four drugs for VL treatment are available. 1) Pentavalent antimonials have been the first-line treatment for over 70 years. This treatment is long (20 to 30 days), is toxic (3–5% deaths due to treatment) and is accompanied by increasing failure rates; for instance in foci of the Bihar state, India, with up to 60% treatment failures, a phenomenon that is assumed to be caused by drug resistance [19]. 2) Miltefosine is the first oral drug against VL and has been recommended as first-line drug in the VL elimination initiative but is teratogenic and is suspected to rapidly give rise to resistance due to its long half-life. 3) Amphotericin B is used in two formulations: “Conventional” Amphotericin B and “Liposomal” Amphotericin B (Ambisome). Treatment with the conventional formulation is long (30 days) and can have life-threatening side effects whereas treatment with the liposomal formulation is shorter, has similar high efficacy and fewer side effects; Yet it is too expensive and complex in its administration for large-scale application in developing countries. However recently, a single-dose Ambisome schedule was found highly effective in India [20]. 4) Paromomycin (PMM) was registered in 2006 in India and is currently being tested in a phase IV trial [2], [21].

#### Vector control

Vector control is an effective tool for controlling VL, as demonstrated in the 1950s in India by malaria control with DDT. Indoor spraying, in particular, reduces sand fly densities, also long-lasting insecticide-treated bed nets and to a minor extent environmental management [22], [23]. However, the recent community intervention trial KalaNet failed to demonstrate an effect of long-lasting insecticide-treated nets on the incidences of *L. donovani* infection and VL [24].

### Mathematical modelling

The first mathematical study of the dynamics of KA employed a deterministic model to explain the observed inter-epidemic periods between 1875 and 1950 in Assam, India [25]. This model was later extended to canine visceral leishmaniasis in Malta, considering three different serological tests for the presence of specific antibody in dogs [26], [27]. In a further extension, the efficacies of various control methods, such as vaccination, killing infected dogs, drugs and insecticides, were investigated by means of sensitivity analyses [28]. A recent modelling paper addressed the question of under-reporting of VL in Bihar, India. [29].

Our model focuses on the transmission dynamics of *L. donovani* in the Indian subcontinent and extends the before-mentioned modelling findings with (i) quantifications of epidemiological parameters for the natural history of infection and transmission dynamics of *L. donovani* and (ii) databased predictions for the effects of different intervention strategies; in particular, the roles of asymptomatic and symptomatic infections are considered.

## Methods

### Model

**Figure 1:**
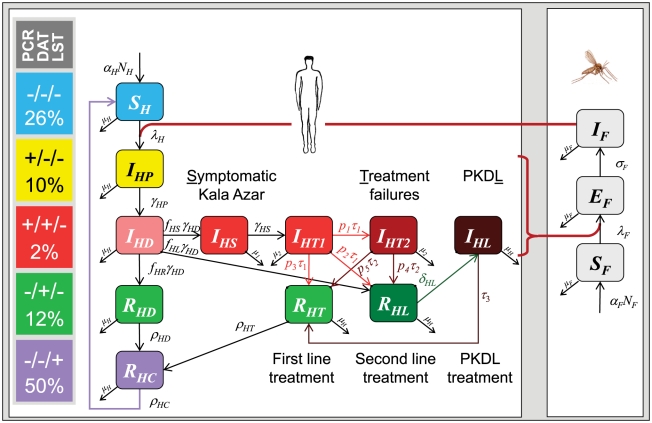
Model for *L. donovani* infection, transmission and control. Compartments represent proportions in humans and vectors, distinguished (vertically) according to their history of infection (defined by diagnostic states). The diagnostics comprised PCR, DAT and LST with combinations shown in the bar on the left margin of the graph. Human hosts are further distinguished (horizontally) by disease and treatment status. *μ_1_* = *μ_H_+μ_K_*; *μ_2_* = *μ_H_+μ_K_+μ_T1_*; *μ_3_* = *μ_H_+μ_K_+μ_T2_*; for further variables and parameters see text, Table 1, Table 2, Table 3 and Table S1 in the Supplement.

The transmission dynamics of *L. donovani* in the Indian subcontinent was modelled deterministically by a system of ordinary differential equations. Figure 1 describes the model graphically, and Table 1, 2 and 3 provide lists of parameters and variables with references. In this section, we describe the basic structure of the model; the full model, considering also possible animal hosts and immuno-compromised humans, is provided in Text S1 with Figure S1 showing the full model.

**Table 1:** Model parameters and variables – sand flies.

	Description	Value	Reference
*N_F_*	No. of vectors per *N_H_* = 100 humans, see below)	527	Estimated, 95% CI (347 to 990)
*I_F_*	Equilibrium prevalence of infectious sand flies	0.5%	[35], [41], [42]
1/*μ_F_*	Life expectancy of sand flies	14 days	[43]
1/*σ_F_*	Sojourn time§ of flies in the latent stage *E_F_*	5 days	[44]
1/*β*	Feeding cycle duration	4 days	[36]
*p_H_*	Probability that a human becomes infected when an infectedfly takes a blood meal on a susceptible person	1	Assumed (correlated with *N_F_*)

**§:** Durations regardless of mortality, i.e., conditional on surviving (e.g. the sojourn time of flies in the latent stage *E_F_* would equal 1/(*σ_F_*+*σ_F_*) if mortality is considered).

**Table 2:** Model parameters and variables – humans.

	Description	Value	Reference
*N_H_*	No. of humans	100	Scaling factor (in relation to *N_F_*)
*S_H_+R_HC_*	Prevalence* of PCR^−^DAT^−^humans	76%	KalaNet data
*I_HP_*	Prevalence* of PCR^+^DAT^−^humans	10%	KalaNet data
*I_HD_*	Prevalence* of PCR^+^DAT^+^humans	2%	KalaNet data
*R_HD_*	Prevalence* of PCR^−^DAT^+^humans	12%	KalaNet data
*I_HS_+I_HT_*	Prevalence* of symptomaticKA	0.015%	KalaNet data
*I_HL_*	Prevalence* of PKDL	0.005%	Model result basedon a fraction of treatment failure *p_2_* = 3%
*R_HC_*	Prevalence* of LST^+^ humans	50%	[31], [32], [33]
1/*μ_H_*	Baseline life expectancy ofhumans	40 years	Assumed
*μ_K_*	Excess mortality rate causedby KA	1/(5 months)	Assumed
*p_F1_*	Probability that a susceptible fly becomes infected when feedingon a human host of type *I_HP_*	0.0125	= *p_F2_*/2 (assumed)
*p_F2_*	Probability that a susceptible fly becomes infected when feedingon a human host of type *I_HD_*	0.025	Estimated, 95%CI (0.012 to 0.038)
*p_F3_*	Probability that a susceptible fly becomes infected when feedingon a human host of type *I_HS_*, *I_HT1_*,*I_HT2_*	1	Assumed
*p_F4_*	Probability that a susceptible fly becomes infected when feedingon a human host of type *I_HL_*	1	Assumed
*f_HS_*	Fraction of asymptomatically infected hosts (*I_HD_*) who will develop symptomatic KA	0.33%	Estimated, 95% CI (0.22% to 0.49%)
*f_HL_*	Fraction of asymptomatically infected hosts (*I_HD_*) who will develop PKDL	0.01%	[9]: 8 cases in22699 personsduring 6 years
*f_HR_*	Fraction of asymptomaticallyinfected hosts (*I_HD_*) who willrecover without showing a symptomatic course ofinfection (→*R_HD_*)	99.77%	= 1−(*f_HS_*+*f_HL_*)
1/*γ_HP_*	Sojourn time^§^ in the early asymptomatic stage *I_HP_*	60 days	[37], [45]
1/*γ_HD_*	Sojourn time^§^ in the late asymptomatic stage *I_HD_*	12 days	Estimated, 95%CI (9 to 15)
1/*γ_HS_*	Duration between diagnosisof KA and onset of treatment	1 day	Conditions in the KalaNet trial
1/*ρ_HD_*	Period^§^ of DAT-positivityin state *R_HD_*	74 days	Estimated, 95%CI (65 to 84)
1/*ρ_HT_*	Period^§^ of DAT-positivityin state *R_HT_*	74 days	= 1/*ρ_HD_* (assumed)
1/*ρ_HC_*	Period^§^ of LST-positivityin state *R_HC_*	307 days	Estimated, 95% CI (260 to 356)

*Prevalences include immuno-compromised humans.

^§^ Durations regardless of mortality.

**Table 3:** Model parameters and variables – treatment.

	Description	Value	Reference
1/*τ_1_*	Duration of first-line KAtreatment	30 days	[21]
1/*τ_2_*	Duration of second-line KA treatment	30 days	[21]
1/*τ_3_*	Duration of PKDL treatment	180 days	[9]
*f_T1_*	Fraction of treatment fatality	5%	Personal communication, MB
*μ_T1_*	Excess mortality rate caused byfirst-line KA treatment	0.00167	= *f_T_τ_1_*
*μ_T2_*	Excess mortality rate caused by second-line KA treatment	0.00167	= *f_T_τ_2_*
*p_1_*	Fraction of KA patients not responding to KA first-linetreatment (conditional onsurviving treatment, 1−*f_T_*)	5% (100% = *p_1_*+*p_2_*+*p_3_*)	[21]
*p_2_*	Fraction of KA patients whoappear to recover under KAfirst-line treatment but willdevelop PKDL (conditional on surviving treatment, 1−*f_T_*)	3% (100% = *p_1_*+*p_2_*+*p_3_*)	Personal communication, MB
*p_3_*	Fraction of KA patientsrecovering during KAfirst-line treatment (conditionalon surviving treatment, 1−*f_T_*)	92% (100% = *p_1_*+*p_2_*+*p_3_*)	= 1−(*p_1_*+*p_2_*)
*p_4_*	Fraction of KA patients whoappear to recover under KAsecond-line treatment but will develop PKDL (conditional on surviving treatment, 1−*f_T_*)	3% (100% = *p_4_*+*p_5_*)	= *p_2_*
*p_5_*	Fraction of KA patientsrecovering during KAsecond-line treatment(conditional on survivingtreatment, 1−*f_T_*)	97% (100% = *p_4_*+*p_5_*)	= 1−*p_4_*
1/*δ_HL_*	Duration until relapse to PKDL	21 months	[9], [46], [47]

For parameters and variables concerning immuno-compromised patients, see Table S1.

The basic Susceptible-Infected-Recovered (SIR) type model was modified with respect to the history of infection, considering five stages in the natural history of infection. These stages were described based on different combinations of three diagnostic markers to categorise people living in endemic areas: PCR (index P, earliest marker for *L. donovani* infection), DAT (index D, antibody response) and LST (index C, suggesting a state of ‘cellular’ immunity). Superscripts “+” and “−” represent positive and negative results, respectively. If the infection proceeded asymptomatically, we assumed that the human host (indicated by subscript H) typically passed through the following five stages:

***S_H_***: *Susceptible stage* (**P^−^D^−^C^−^**). Hosts are negative for all three markers and can become infected in the future.

***I_HP_***: *Early asymptomatic*, *infectious stage* (**P^+^D^−^C^−^**). The parasite can be detected by PCR, but there is not yet any humoral or cellular response in the host.

***I_HD_***: *Late asymptomatic*, *infectious stage* (**P^+^D^+^C^−^**). Hosts are still PCR-positive and antibodies can be detected by DAT.

***R_HD_***: *Early recovered stage* (**P^−^D^+^C^−^**). The parasite cannot be detected anymore, but hosts are DAT-positive and not yet LST-positive.

***R_HC_***: *Late recovered stage* (**P^−^D^−^C^+^**). Hosts are DAT-negative but still LST-positive and assumed to be protected against re-infection.

#### Natural history of infection in humans

Humans are born as susceptibles (*S_H_*) at a per capita birth rate *α_H_*. We assume a constant population size *N_H_* based on constant birth and death rates (*μ_H_*). Susceptibles are infected at rate *λ_H_*, that depends on 1) the biting rate *β* of sand flies, 2) the probability of becoming infected when being bitten by an infectious sand fly (*p_H_*) and 3) the number of infectious sand flies per human (*I_F_* per *N_H_*).

After infection, humans enter the early asymptomatic stage *I_HP_* and become PCR-positive in peripheral blood. Seroconversion occurs at rate *γ_HP_* into the late asymptomatic stage *I_HD_*, which is characterised by the onset of DAT-positivity. If not dying, humans remain in this asymptomatic stage for 1/*γ_HD_* days. Infection with Leishmania parasites proceeds in most cases asymptomatically, with only a minor fraction of cases subsequently developing KA. A major fraction *f_HR_* does not develop disease, becomes PCR-negative in the early recovered stage *R_HD_*, and develops LST-positivity (*R_HC_*) at rate *ρ_HD_*. A remaining fraction of 1−*f_HR_* asymptomatic infections is split into a tiny fraction *f_HL_* of putatively recovering humans who develop a state of PCR-negativity in peripheral blood, while still harboring a non-detectable number of parasites; this is denoted *R_HL_*, from where relapse to PKDL (*I_HL_*) follows. A fraction (*f_HS_*) develops symptomatic KA (*I_HS_*) whilst staying PCR-positive.

#### Therapy

The fraction *f_HS_* developing symptomatic KA (*I_HS_*) is eligible for treatment. If not dying, these patients receive first-line treatment (*I_HT1_*) on average after 1/*γ_HS_* days. First-line treatment (in state *I_HT1_*) is given for 1/*τ_1_* days. A proportion *p_3_* of patients clears parasites under first-line treatment, recovers (→*R_HT_*), and finally becomes LST-positive (→*R_HC_*) like those with asymptomatic infections. The remaining proportion 1−*p_3_* of patients represents the treatment failures that are split into a PCR-positive proportion of *p_1_* KA patients receiving second-line treatment (*I_HT2_*) and a proportion of *p_2_* patients putatively recovering into a state of PCR-negativity. The second proportion still harbours a non-detectable number of parasites (*R_HL_*, from where relapse to PKDL, *I_HL_*, will follow).

Second-line treatment (in state *I_HT2_*) is given for 1/*τ_2_* days. As with first-line treatment, a proportion *p_5_* of patients under second-line treatment recovers (→*R_HT_*) and becomes LST-positive (→*R_HC_*). The remaining proportion of *p_4_* patients recovers putatively into a state of PCR-negativity (*R_HL_*) from which, again, relapse to PCR-positivity and PKDL (*I_HL_*) follows, for those who do not die on average after 1/*δ_HL_* days. All PKDL patients (*I_HL_*) are treated until full recovery (→*R_HT_*→*R_HC_*).

Excess mortality 1) affects KA patients whose mortality rate increases the baseline mortality rate *μ_H_* to *μ_1_* = *μ_H_+μ_K_* and 2) occurs under treatment because of drug toxicity. Five percent of patients die because of first-line or second-line treatment, increasing the mortality rate to *μ_2_* = *μ_H_+μ_K_+μ_T1_* or *μ_3_ = μ_H_+μ_K_+μ_T2_*. LST-positivity (suggesting a state of cellular immunity) may remain over a period of 1/*ρ_HC_* days, after which recovered humans (*R_HC_*) again become negative for all diagnostic markers (→*S_H_*).

#### Vectors

We considered sand flies in the susceptible (*S_F_*), latent (*E_F_*, not infectious), or infectious (*I_F_*) stages. Flies can become infected by blood meals taken on infectious humans (*I_HP_*, *I_HD_*, *I_HS_*, *I_HT1_*, *I_HT2_*, *I_HL_*). The infection rate *λ_F_* of flies is determined by the following: 1) the biting rate *β*, 2) the infection probabilities of flies dependent on the infection status of the hosts (*p_F1_* to *p_F4_*, see Table 2) and 3) the numbers of infectious hosts.

We assumed that each blood meal of a susceptible sand fly leads to a sand fly infection if taken from a symptomatically infected human (*p_F3_* = 100% for KA patients *I_HS_*, *I_HT1_*, *I_HT2_* and *p_F4_* = 100% for PKDL patients *I_HL_*). For a sensitivity analysis, see Results.

The probability that a fly becomes infected when feeding on asymptomatically infected hosts of type *I_HP_* or *I_HD_* was estimated with the model as follows. The infection probability *p_F2_* of late asymptomatically infected humans (*I_HD_*) was estimated, whereby we assumed that the infectivity of hosts increases monotonically from the time of infection until the late asymptomatic state *I_HD_*. We defined an intermediate infection probability for flies originating from hosts in the early asymptomatic state *I_HP_* as *p_F1_* = *p_F2_*/2.

### Data

#### KalaNet data

The model was fitted to data from the KalaNet project (KALANET, ClinicalTrials.gov NCT00318721), a community intervention trial in India and Nepal to investigate the effectiveness of insecticide-treated bed nets. The KalaNet trial was conducted between 2006 and 2008. Around 35,000 cases of VL have been reported during this period by the Government of India, Ministry of Health and Family Welfare; thus, the prevalence of VL during the KalaNet trial was lying almost in between the official numbers of 10,000 and 75,000 cases, respectively, after 1970 [30]. In the KalaNet trial, consecutive blood samples were collected in 26 highly endemic villages from 17,662 people older than two years. These blood samples were used to diagnose infection of humans, by assessing seroconversion with the direct agglutination test (DAT). Passive and active detection for VL cases were conducted until May 2009. In addition, blood samples for PCR analysis were collected (a) in a random sub sample of all KalaNet villages from 625 people between 15 and 40 years of age and (b) from all inhabitants older than 3 years in one Nepalese KalaNet village (284 people).

Interventions in the KalaNet trial did not show an effect on VL incidence and seroconversion; thus, we included data from interventions and control clusters in our analyses. The KalaNet data showed a prevalence of 12% PCR-positive (from 1,923 measurements on 909 individuals in total) and 14% DAT-positive individuals (from 43,171 measurements on 17,662 individuals in total). An intersection of 2% was positive for both markers (from 1,899 measurements on 893 individuals in total). Hence, 76% of the population was negative for PCR and DAT. Confidence intervals for these prevalences were not computed, as the large sample size would suggest an unrealistically high precision. No LST data were available in the KalaNet study. The average incidence of symptomatic VL was 0.27% per year, with an estimated prevalence of 0.015% (15 cases per 100,000). Definition for symptomatic infection was adopted from the KalaNet project: People with fever lasting for two weeks or more were examined by a physician and tested with a rapid diagnostic test for VL [24]. Prevalence of *Leishmania* infection in sand flies as determined by PCR was 0.5% in six Nepalese clusters.

#### Leishmanin skin test (LST)

The before-mentioned proportion of 76% of humans who are negative for PCR and DAT includes 1) humans who either have never been infected (susceptible newborns: *α_H_ N_H_*→*S_H_*), 2) humans who recovered from infection (*R_H_*→*R_HC_*), or 3) humans who have lost LST-positivity thereafter (*R_HC_*→*S_H_*). We assumed that LST-positivity could be lost because the consecutive PCR and DAT results of several KalaNet participants showed re-infection (first positive, then negative and then positive again). The prevalence of LST-positive humans (*R_HC_*) was taken from published studies on LST in Nepal [31], India [32] and Bangladesh [33], adopting an average sensitivity of the LST of about 50% [33]. With these estimates, the prevalence of 76% humans who are negative for PCR and DAT can be split into a proportion of 50% LST-positive (*R_HC_*) and 26% susceptible (*S_H_*). The effects emerging from variations in these proportions will be discussed (see Discussion).

#### PKDL

There was no active detection for PKDL cases in the KalaNet trial. The proportion of treatment failures among KA patients under first- and second-line treatments who will develop PKDL via a putatively recovered stage (*I_HT1_* and *I_HT2_*→*R_HL_*→*I_HL_*) is roughly 3% in India/Nepal (MB, personal communication), which yields, together with a tiny proportion of asymptomatically infected humans who develop PKDL without treatment (*I_HD_*→*R_HL_*→*I_HL_*) [9], a prevalence of 0.5 PKDL cases per 10,000 (see Results). Putatively recovered KA patients develop PKDL after 21 months on average [9].

#### Treatment

First- and second-line treatment lasts about 1 month for KA patients and about 6 months for PKDL patients. For KA patients surviving treatment, a fraction of treatment failure of 5% is assumed [9], [21]. The effects emerging from variations in these parameters were considered in a sensitivity analysis (see Results).

### Parameter estimation

Model solutions were numerically computed using the software Matlab Version 7.120635 (R2011a) by a Runge-Kutta algorithm with variable step size (procedure ode15s). Parameters were estimated by fitting the model to observations from the KalaNet project (DAT- and PCR-positivity, symptomatic VL and sand fly infection) and information from the literature (LST-positivity, prevalence of HIV and prevalence of HIV-infected among symptomatic VL).

We used the model to estimate the following eight parameters:

1. *N_F_* determining the density of sand flies (*N_F_* per *N_H_*)
2. 1/*γ_HD_* determining the sojourn time in the late asymptomatic stage *I_HD_*
3. 1/*ρ_HD_* determining the period of DAT-positivity in state *R_HD_*
4. 1/*ρ_HC_* determining the period of LST-positivity in state *R_HC_*
5. *p_F2_* determining the probability that a fly becomes infected when feeding on an asymptomatically infected human host in state *I_HD_*
6. *f_HS_* determining the fraction of asymptomatically infected hosts (*I_HD_*) who will develop KA
7. *f_VS_* determining the fraction of immuno-compromised and asymptomatically infected hosts (*I_VD_*) who will develop symptomatic KA (see Table S1)
8. *η* determining the HIV infection rate (see Table S1)

The parameter vector 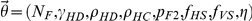 was estimated by Maximum Likelihood with likelihood function
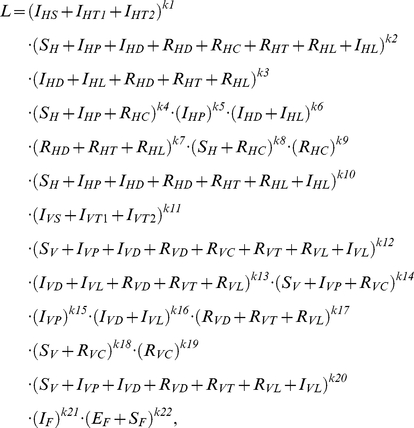
where by exponents k1 to k22 represent sample sizes observed in the KalaNet trial or taken from the literature (for references see Table 1, 2, 3 and Table S1). These exponents are partially not integer values, because sample sizes for the estimation had to be back calculated considering the prevalence of HIV-positive hosts, yielding
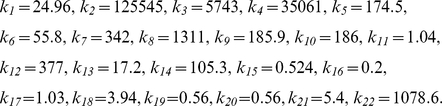



The likelihood was maximized by minimizing the negative log-Likelihood −ln(L), using Matlab procedure fminsearch. Confidence intervals were computed by the profile likelihood [34], i.e. the parameter of interest was minimised (lower confidence limit) or maximised (upper confidence limit) conditional on that the likelihood worsens only according to *χ* ^2^/2 with 1 degree of freedom, i.e. 3.84/2, whereby the other 7 parameters may vary as nuisance parameters. Parameter values, estimates and confidence intervals are shown in Tables 1, 2, 3 and Table S1.

## Results

### Parameter estimates

The parameter estimates (Table 1, Table 2, Table 3 and Table S1) originate from fitting the model to data of which prevalences have been estimated as described in the Methods section were as follows 26% of the human population (*S_H_*) were negative for all three markers of PCR, DAT and LST (P^−^D^−^C^−^), 10% (*I_HP_*) were PCR-positive (P^+^D^−^C^−^), 2% (*I_HD_*+*I_HS_*+*I_HT1_*+*I_HT2_*+*I_HL_*) were PCR- and DAT-positive (P^+^D^+^C^−^), 12% (*R_HD_*+*R_HT_*+*R_HL_*) were DAT-positive (P^−^D^+^C^−^) and 50% (*R_HC_*) were LST-positive (P^−^D^−^C^+^). Model-based rates and durations in the natural history of infection were estimated from the prevalences for humans as follows.

Humans remained on average 1/(*λ_H_*+*μ_H_*) = 150 days in the susceptible state (*S_H_*) before they became infected. After infection, PCR-positivity lasted on average for 1/(*γ_HP_*+*μ_H_*)+1/(*γ_HD_*+*μ_H_*) = 72 days (approx. 95% CI: 69 to 75 days) and overlapped with DAT-positivity, which lasts for asymptomatic individuals for 1/(*γ_HD_*+*μ_H_*)+1/(*ρ_HD_*+*μ_H_*) = 86 days (approx. 95% CI: 74 to 99 days). For an overlap period of about 1/(*γ_HD_*+*μ_H_*) = 12 days, asymptomatic individuals were positive for both markers (late asymptomatic state *I_HD_*). This means that individuals without symptomatic disease need on average about 146 days to develop LST-positivity after a PCR-positive finding. LST-positivity lasted on average 1/(*ρ_HC_*+*μ_H_*) = 300 days (approx. 95% CI: 255 to 348 days). As population turnover alone is not sufficient to yield a prevalence of 26% susceptibles (negative for all three markers: P^−^D^−^C^−^), loss of LST-positivity must be assumed to explain these, together with a prevalence of 50% LST-positive individuals in the population.

Taken these durations together, humans with an asymptomatic course were infected on average every 596 days ( = 150 days *S_H_*+60 days *I_HP_*+12 days *I_HD_*+74 days *R_HD_*+300 days *R_HC_*). As heterogeneities are not taken into account, one might crudely assume that humans become infected every one to three years.

Given a 0.5% prevalence of infected sand flies [35] and the above-mentioned prevalences, the probability *p_F2_* that a sand fly is infected during a blood meal on an asymptomatic host (*I_HD_*) was estimated at 2.5% with 95% CI (1.2% to 3.8%). This estimate is robust against changes in the infection probability for flies feeding on symptomatic hosts, which was assumed to be *p_F3_* = *p_F4_* = 100%. A sensitivity analysis in which *p_F3_* and *p_F4_* were reduced to 10% had no relevant effect on the estimate for *p_F2_*, which then decreased from 2.5% to 2.3%. The reason for this minor contribution of symptomatically infected hosts to the overall transmission is simple: their prevalence is too low compared to the abundance of asymptomatically infected hosts. The estimates reported so far are correlated with the time between blood meals of sand flies (assumed with 1/*β* = 4 days [36]) and the ratio *N_F_*∶*N_H_* of vectors to humans (estimated at 527∶100, with 95% CI*_NF_* (347 to 990)).

### Intervention

We investigated interventions recommended by the VL elimination program, including treatment-related control strategies, such as early case detection or treatment optimisation. We also considered vector-related control strategies, such as breeding site control, indoor spraying or use of bed nets, to explore possibilities and constraints in VL control.

#### Treatment-related interventions

**Figure 2:**
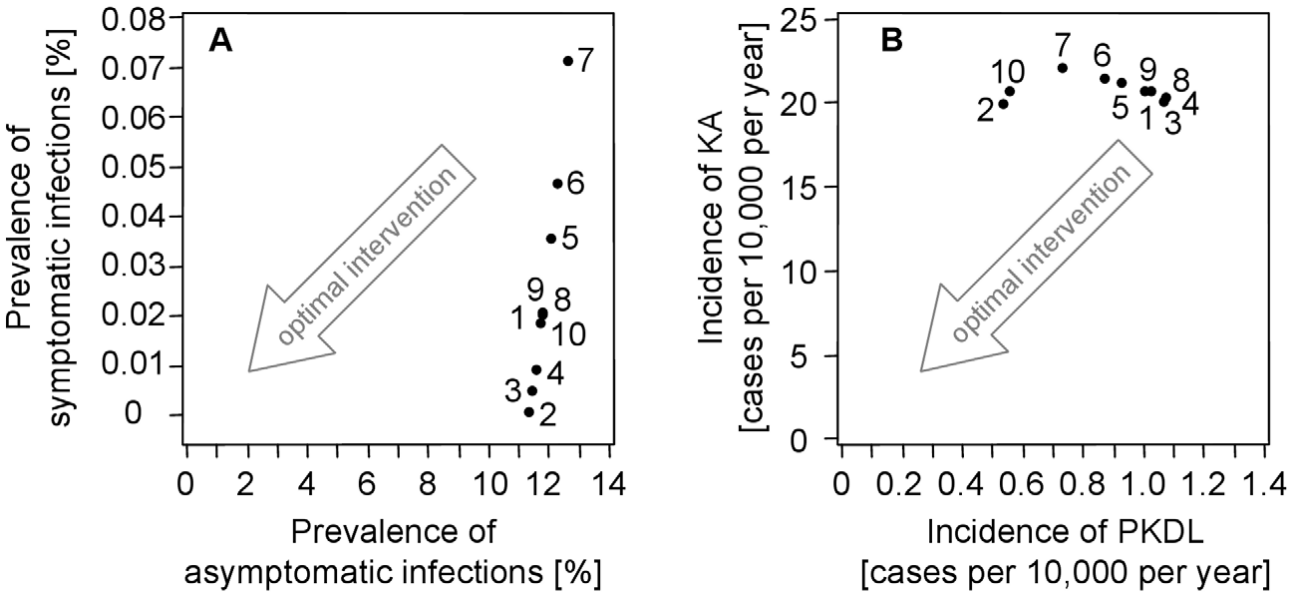
Treatment-related interventions. Sensitivity analyses of equilibrium solutions to the effects of seven intervention parameters (A) on the prevalence of symptomatic (*I_HS_*, *I_HT1_*, *I_HT2_*, *I_HL_*) and asymptomatic infections (*I_HP_*, *I_HD_*) and (B) on the incidences of KA and PKDL. The ten scenarios refer to ten parameter combinations as shown in Table 4. The default scenario 1 used parameter values as obtained from model calibration. The duration of treatment (parameters *τ_1_*, *τ_2_*, *τ_3_*) varied in scenarios 3 and 4; early case detection (1/*γ_HS_*) varied in scenarios 5, 6, and 7; and treatment efficacy (*f_T_*, *p_1_*, *p_2_*) varied in scenarios 8, 9 and 10. Scenario 2 represents a best-case scenario, using over-optimistic assumptions for all parameters. The default scenario 1 was compared to more pessimistic intervention parameters in scenarios 5, 6 and 7 and to more optimistic intervention parameters in scenarios 2, 3, 8, 9 and 10. As illustrated by the diagonally proceeding arrow, an optimal intervention would reduce the prevalence and incidence in both dimensions (see Fig. 3 and Table 5 for vector-related interventions).

We used the model to predict how different treatment regimens and early case detection affect VL prevalence and incidence. Early case detection, e.g., due to improved diagnosis, reduces the period of symptomatic KA before the start of treatment. The existing drugs vary in their treatment durations (from 30 days for antimonials and conventional Amphotericin B to 5 days for Ambisome) and in their recovery rates (from 99% for conventional Amphotericin B to 91% for Ambisome). We used seven model parameters to study the effects of the different treatment-related interventions, including the following: duration of first-line, second-line and PKDL treatments (*τ_1_*, *τ_2_*, *τ_3_*), duration of KA before treatment (determined by *γ_HS_*), the fraction of treatment fatality (*f_T_*) and treatment failures leading to KA (*p_1_*) or PKDL (*p_2_*). Sensitivity analyses were performed in ten different intervention scenarios (see Table 4), and equilibrium solutions are shown in Fig. 2.

**Table 4:** Parameter combinations of treatment-related interventions.

	Scenario									
Parameter	Default 1	2	3	4	5	6	7	8	9	10
Duration first-line treatment 1/*τ_1_* (days)	**30**	**1**	**5**	**5**	30	30	30	30	30	30
Duration second-line treatment 1/*τ_2_* (days)	**30**	**1**	**5**	**5**	30	30	30	30	30	30
Duration PKDL treatment 1/*τ_3_* (days)	**180**	**1**	**30**	**180**	180	180	180	180	180	180
Early case detection 1/*γ_HS_* (days)	**1**	**1**	1	1	**42**	**90**	**365**	1	1	1
Treatment fatality *f_T_* (%)	**5**	**0**	5	5	5	5	5	**0**	5	5
Treated fraction leading to retention of KA *p_1_* (%)	**5**	**0**	5	5	5	5	5	5	**0**	5
Treated fraction leading to relapse into PKDL *p_2_* (%)	**3**	**0**	3	3	3	3	3	3	3	**0**

Ten different scenarios were considered for sensitivity analyses of the equilibrium solutions to the effects of seven treatment-related intervention parameters.

Fig. 2A shows that the different treatment regimens can only reduce the prevalence of symptomatic infections, with almost no effect on the prevalence of asymptomatic infections. The default prevalence of symptomatic infections of 0.02% (scenario 1) was not influenced much by the treatment fatality rate or by the failure rates under KA and PKDL treatments (scenarios 8, 9 and 10). A prolonged period of symptomatic KA before treatment increased the prevalence of symptomatic infections (scenarios 5, 6 and 7). The prevalence of symptomatic infections could be reduced to 0.01% if successful KA treatment does not require a treatment duration of 30 but 5 days (scenario 4); a further reduction to 0.004% could be achieved by additionally reducing the duration of successful PKDL treatment from 180 to 30 days (scenario 3). Even under overoptimistic assumptions for all treatment parameters, the treatment does not seem to be capable of reducing the prevalence of symptomatic infection below 0.001% (scenario 2).

Fig. 2B shows the influence of the different treatment regimens on the incidences of KA and PKDL. Improvements in treatment could reduce the incidence of PKDL but have only a minor effect on the incidence of KA. In a best-case scenario without any treatment failures developing PKDL, the incidence of PKDL could be reduced from about 1 case per 10,000 person-years (scenario 1) to 0.6 cases per 10,000 person-years (scenarios 2, 10) when there is no history of KA. A delay between case detection and onset of treatment could only marginally lower the incidence of PKDL but slightly increased the incidence of KA (scenarios 5, 6 and 7). In these scenarios, more KA patients died without having received treatment; i.e., the lower incidence of PKDL must be attributed to KA deaths.

These results suggest that transmission of KA is predominantly driven by asymptomatically infected hosts who are not eligible for treatment. Treatment can reduce the prevalence of symptomatic disease, but the incidence of KA still remains on comparable levels. For a given treatment efficacy, shorter treatment durations reduced the patients' treatment burden but had little effect on the overall intensity of transmission.

#### Vector-related interventions

**Figure 3:**
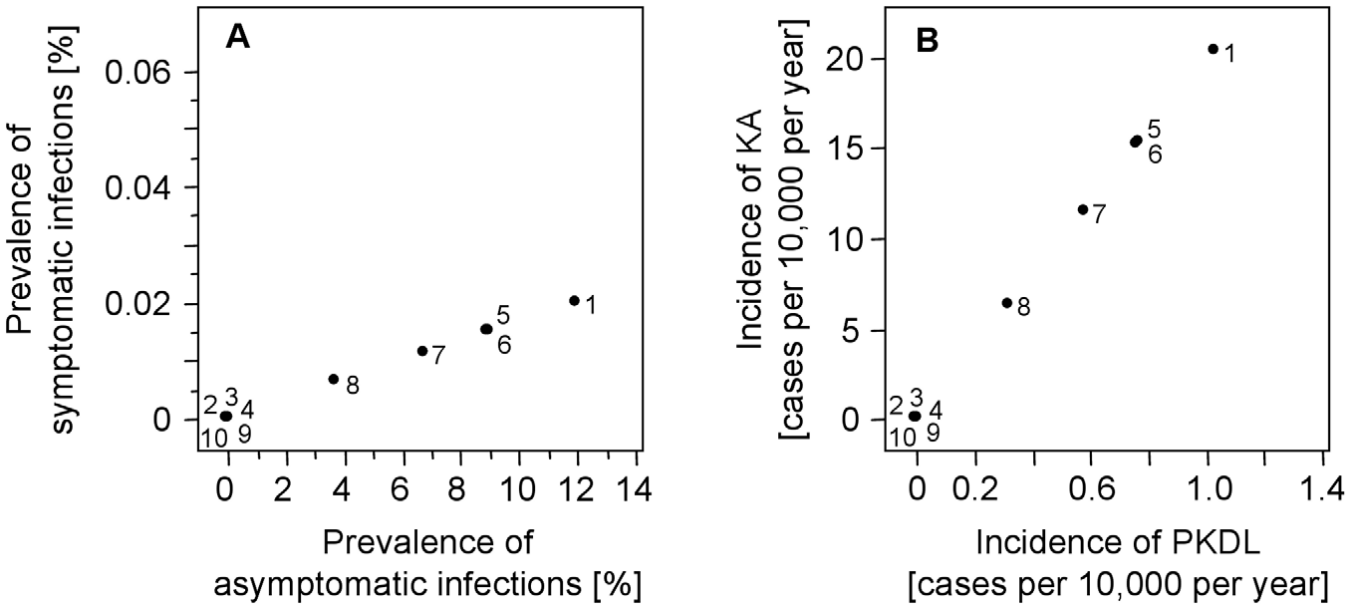
Vector-related interventions. Sensitivity analyses into the effects of vector control: (A) on the prevalence of symptomatic and asymptomatic infections and (B) on the incidences of KA and PKDL. The scenarios refer to ten parameter combinations shown in Table 5. The default scenario 1 uses parameter values as obtained from model calibration. Vector population size *N_F_* varied in scenarios 2 and 5; the flies' life expectancy 1/*μ_F_* varied in scenarios 3 and 6; and their feeding cycle duration 1/*β* varied in scenarios 4 and 7. Scenarios 8, 9 and 10 represent combinations thereof.

We selected three model parameters to study the effects of breeding site control, indoor spraying and use of bed nets, the effects of which were interpreted in terms of the model as follows. Breeding site control was assumed to reduce the vector population size *N_F_*, indoor spraying was assumed to shorten the life expectancy 1/*μ_F_* of sand flies, and the universal use of untreated bed nets was assumed to lower the contact rate *β*. Sensitivity analyses were performed on these three model parameters in ten different intervention scenarios (see Table 5), assuming that the interventions were applied equally to all humans. Equilibrium solutions are shown in Fig. 3.

**Table 5:** Parameter combinations of vector-related interventions.

	Scenario									
Parameter	Default 1	2	3	4	5	6	7	8	9	10
No. of vectors (*N_F_*) per *N_H_* = 100 humans	**527**	**100**	527	527	**300**	527	527	**300**	**300**	527
Life expectancy of sand flies 1/*μ_F_* (days)	**14**	14	**7**	14	14	**11**	14	**11**	14	**11**
Feeding cycle duration 1/*β* (days)	**4**	4	4	**8**	4	4	**6**	4	**6**	**6**

Ten different scenarios were considered for sensitivity analyses of the equilibrium solutions to the effects of three vector-related intervention parameters.

Figs. 3A and B demonstrate that, in contrast to treatment, vector-based interventions reduce the prevalence of symptomatic and asymptomatic infections, which implies that the incidences of KA and PKDL were also reduced. VL could even be eliminated by decreasing the vector density from *N_F_* = 5.27 to 1 fly per human host, by halving the sand flies' life expectancy from 1/*μ_F_* = 14 days to 7 days, or by increasing their feeding cycle duration from 1/*β* = 4 days to 8 days (scenarios 2, 3 and 4). Combinations of feeding cycle duration of, for example, 6 days with a vector density of 3 flies per human, or a life expectancy of 11 days also lead to elimination (scenarios 9 and 10). Changes in parameter values had an almost linear effect on prevalence and incidence, so the effect of intermediate parameter values could be crudely derived by interpolation (scenarios 5, 6, 7, 8).

**Figure 4:**
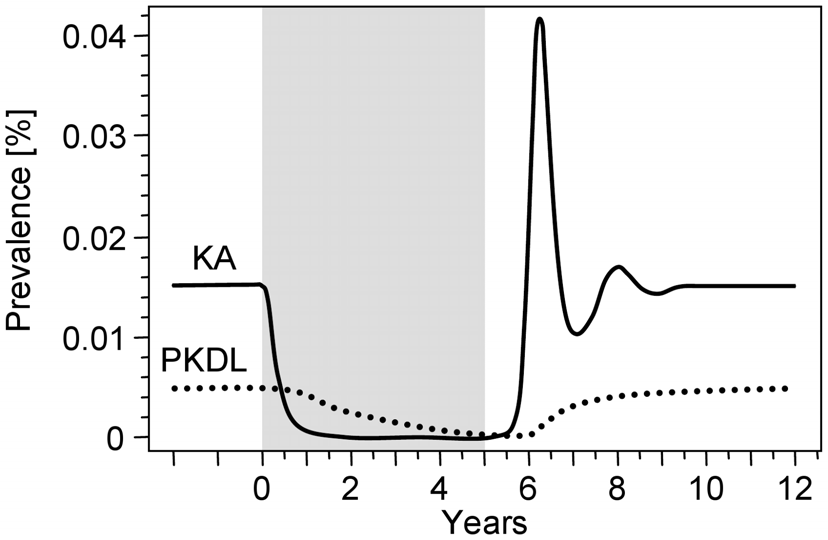
Time-dependent effect of reducing the contact rate. We assumed that the feeding cycle duration of the sand fly was doubled by the intervention from 1/*β* = 4 days to 8 days (scenario 4 in Fig. 3 and Table 5). The intervention lasted for 5 years (grey box). The solid curve shows the prevalence of KA and the dotted line the prevalence of PKDL.

The equilibrium solutions shown in Fig. 3 and Table 5 should not be misleading in the sense that the dynamic properties of transmission and control can be neglected. We therefore illustrated the effect of reducing the contact rate (scenario 4 of Fig. 3 and Table 5) in its time-dependent solution as shown in Fig. 4. The prevalences of KA and PKDL could be effectively reduced by means of vector control, but the effect on PKDL was delayed because putatively recovered KA patients may relapse to PKDL after months to years. This finding implies that an effective reduction in the prevalence of PKDL will become apparent only after years. After the intervention is stopped and the biting rate reaches default values, the few remaining infectious people can initiate a new outbreak of the disease.

## Discussion

Visceral Leishmaniasis is a neglected, life-threatening disease affecting the poorest of the poor, as this vector-borne disease is strongly linked to poor housing [1]. Leishmaniasis has received more attention in light of the regional VL elimination program. A deterministic compartmental model was developed to estimate parameters for *L. donovani* transmission and to optimise intervention success. Uncertainties originating from a high-dimensional parameter space and resulting correlations between parameters have been explored by means of sensitivity analyses (see section on sensitivity analyses at the end of Discussion). For instance, varying the life expectancy of humans from 40 years to between 30 and 60 years did not change the estimates in a relevant way. Furthermore, uncertainties can originate from spatial heterogeneities, which are not included in this modelling approach (see Discussion about clustered infection in the following section). Parameter estimates (see Table 1, Table 2, Table 3 and Table S1) were derived from fitting the model to database average prevalences, under the assumption of homogeneous spread of the parasites within the human and fly populations.

### Natural history of infection

The transmission dynamics of *L. donovani* are rather slow, mainly due to the long incubation period and the potentially long persistence of parasites in infected humans. Parasite DNA can be detected in infected human hosts for two to three months; after seroconversion, antibodies can be detected for about the same period of time in those humans who do not evolve to clinical disease. These periods overlap for a few weeks only, when both parasites and antibodies are detectable in peripheral blood. Few infected humans become clinically sick. The majority of infected hosts are estimated to become LST-positive five to six months after infection, without showing any sign of disease. LST-positivity, which is assumed to represent a state of protective cellular immunity, is estimated to persist for about one year. Without loss of LST-positivity, the model would lead under realistic birth and death rates of humans to over 90% LST-positive individuals in the population.

For analysing the process of human recovery, published studies involving the LST were used to estimate the duration of the cellular immune response based on LST-positivity. Previous studies reported prevalences of positive LST results among recovered KA patients (who are expected to be LST-positive) ranging from 30% to 80% [33]. These studies suggested that the lowness and variability of LST sensitivity is affected by the leishmanin antigen suspension chosen. Because an antigen based on South Asian *L. donovani* is not available, all LST data refer to *L. infantum* or *L. major* antigens. There is extensive cross-reactivity of patient responses to heterologous *Leishmania* species, but as the variable results underscore, there is a need for better standardisation and documentation of sensitivity and stability of leishmanin antigens to obtain more reliable data on the state of cellular immunity. Effects associated with differing proportions of LST-positive individuals are explored in the section on sensitivity analyses below.

Apart from problems with the antigen (see above), there are in principle two possibilities to explain a proportion of only 50% LST-positive individuals (cf. before: >90% LST-positive individuals would be expected under the assumption of life-long LST-positivity). These two explanations depend on how infection spreads, as follows: 1) If infection is clustered (e.g., within-household transmission predominates due to a low rate of transmission between households) then life-long LST-positivity may exist on the level of the individual, and a low, ‘average’ LST-prevalence in the population must result from the demographic process, i.e., it must result from deaths of LST-positive individuals and the births of individuals who are LST-negative. 2) If infection is not clustered, but spreads homogeneously within the human population, then a low LST-prevalence in the population must involve loss of the ‘individual’ LST-positivity (as opposed to option 1 where the ‘average’ LST-positivity gets lost). As the birth rate is not sufficient to explain a prevalence of 50% LST-positive individuals (see above), we proceeded with the assumption of loss of LST-positivity. Under this assumption, humans in active *L. donovani* transmission foci were infected every one to three years.

A considerable loss of LST positivity has been reported from an endemic VL focus in Ethiopia [37]. There, the authors concluded that the presumption of a life-long positive LST reaction may only hold true under circumstances of continued exposure to infected sand flies or subclinical infection.

### Treatment

A short and effective treatment regimen or early case detection can reduce the prevalence of KA but has almost no effect on the incidence of the disease (Fig. 2 and Table 4). Treatment is thus an intervention that benefits the individual but not the population. The reason why treatment is not a measure to control the transmission of infection must be attributed to many asymptomatically infected hosts who are - despite a low infectivity for flies - responsible for the majority of parasite transmissions from humans to sand flies. As demonstrated by the sensitivity analysis shown in Fig. 2 and Table 4 (scenario 2), a beneficial effect of treatment on transmission cannot even be expected under over-optimistic assumptions; for instance, minimal duration of treatment and time until treatment start with maximum treatment efficacy. The KA elimination program aims for less than 1 case per 10,000. Even under over-optimistic treatment assumptions, this target would not be reached; the model suggests that elimination of VL would not succeed if intervention was based on treatment alone. The contribution of asymptomatic infections on transmission may, however, be region and strain specific, as apparent from ratios of asymptomatic infections to KA cases ranging from 6∶1 in Brazil to 50∶1 in Spain [38].

### Vector-related interventions

The current vector-related interventions comprise irregular indoor spraying and exposure prophylaxis, such as untreated bed nets. As summarised by [30], indoor insecticide spraying during the Indian National Malaria Eradication Program between 1958 and 1970 had a drastic impact on transmission of *L. donovani*. No VL cases were reported from the state of Bihar during that period. However, within months after the program was terminated, the first cases of KA re-appeared and at the end of 1970, a VL epidemic struck Bihar. In 1992, another eradication program with indoor residual DDT spraying resulted in a sharp decline from 1993 until 1999.

Whereby treatment-related interventions only reduce symptomatic infection, vector control is also efficient against asymptomatic infection (Fig. 3 and Table 5). The model predicted that VL may be eliminated by reducing the vector density by 80%, which seems to provide serious options for the VL elimination program (results not shown). Prospects of successful elimination can also be expressed by the moderate basic reproduction number which this model predicts with *R_0_* = 3.94 (see Text S1).

As found during the KalaNet study, the use of treated bed nets may not provide this efficacy. Indoor density of *P. argentipes* is reported in the study to be reduced by 25% in villages that used treated nets compared with control villages. This intervention reduced the annual incidence of VL at best to 18.8 cases per 10,000, which is far from the elimination target of less than one case per 10,000. The authors hypothesised that a substantial fraction of *L. donovani* transmission occurring outside the house may explain the results obtained in the trial [24]. However, two other studies in the Indian subcontinent showed a much higher reduction in the sand fly density by means of vector-related interventions [22], [39].

Despite the fact that vector control has the potential to effectively reduce *L. donovani* transmission, it must be complemented by the treatment of KA and PKDL cases. The latter can substantially maintain a reservoir of infection, which can initiate a new outbreak of disease after vector control is stopped. As the natural history of *L. donovani* transmission is a rather slow process where parasites can persist in humans over a long period, interventions will show an effect after years, not months, as demonstrated in Fig. 4. PKDL cases may occur after years, and their final recovery may take months or years. If vector control is stopped before parasites are eliminated in the human hosts, then emerging PKDL cases can initiate a new outbreak of disease as soon as there are enough sand flies available for transmission. Thus, active case detection and effective short-course treatment of PKDL combined with effective vector control are required for VL elimination.

### Sensitivity analyses (see also Text S1)

To determine the role of LST-positive individuals (*R_HC_*), the default assumption of 50% LST-positive individuals was varied between 30% and 70% (76% is the maximum possible value because 24% of the human population belong to other diagnostic categories, see above). A low equilibrium prevalence of LST-positive individuals results from a lower infection rate (or lower values of correlated parameters, such as the biting rate or the number of vectors), and a high prevalence of LST-positive individuals can result from a higher infection rate (or higher values of correlated parameters, such as the biting rate or the number of vectors). The only parameter estimate that was affected within the natural history of infection by this variation of the prevalence of LST-positive individuals was the rate of loss of cellular immunity (*ρ_HC_*). The estimate of 1/*ρ_HC_* = 307 days of LST-positivity with the 95% confidence interval between 260 and 356 days (see Table 2) does not support the assumption of a life-long cellular immunity based on life-long LST-positivity (in technical terms, life-long LST-positivity would lead to an excessive accumulation of individuals in state *R_HC_*, which is not consistent with the data).

The effects of different prevalences of infected sand flies were also analysed, as the prevalence of infected sand flies may vary regionally. The default assumption of *I_F_* = 0.5% infected sand flies is based on data from Nepal, whereas higher prevalences have been observed in India. A higher prevalence of infected sand flies results from a lower number of flies (*N_F_*) or from a higher infection probability originating from asymptomatic hosts (*p_F2_*).

We assumed that the period of DAT-positivity is the same for symptomatic and for asymptomatic cases (1/*ρ_HD_* = 1/*ρ_HT_* = 74 days). The estimate of *ρ_HD_* (DAT-positivity in asymptomatic cases) is robust against the assumption that DAT-positivity may last longer in symptomatic cases [40]. In that investigation antibody persistence in symptomatic cases was suggested to last on average about 4 years. Such an increase, however, can be completely compensated in the model by reducing the period of DAT-positivity in asymptomatic cases from 74 to 69 days, showing that the model behaves robust against changes in these assumptions.

A full factorial sensitivity analysis is provided in Fig. S2 in the Supplement.

### Conclusions

Our simulation results show that transmission of *L. donovani* is predominantly driven by asymptomatically infected hosts who are not eligible for treatment. Treatment can reduce the prevalence of symptomatic disease, but the incidence of KA remains on similar levels because of an unchanged intensity of transmission. In contrast to treatment-related interventions, vector-related interventions have the potential to reduce the prevalence of asymptomatic infections and thus are the intervention of choice from an epidemiological perspective. Vector control, however, should be combined with treatment, as PKDL cases can act as reservoirs of infection. This reservoir function originates from the long period of nearly two years on average during which putatively recovered KA patients develop PKDL.

## Supporting Information

Figure S1 **Full model.** In addition to Fig. 1, this diagram shows the compartments of humans coinfected with HIV. HIV was modelled independently of infection with *L. donovani* and emerged with rate *η* from all human compartments.
(TIF)

Figure S2 **Sensitivity analysis.** Sensitivity analysis on the effects of parameter variations on the stationary solutions of the model. The scatter plot matrix lists in lines/columns 1 to 8 the eight estimated parameters (*N_F_*, *p_F2_*, *f_HS_*, *f_VS_*, *η*, 1/*γ_HD_*, 1/*ρ_HD_*, 1/*ρ_HC_*), and in lines/columns 9 to 33 the stationary solutions of the model variables (*S_H_*, *I_HP_*, *I_HD_*, *R_HD_*, *R_HC_*, *I_HS_*, *I_HT1_*, *R_HT_*, *I_HT2_*, *R_HL_*, *I_HL_*, *S_V_*, *I_VP_*, *I_VD_*, *R_VD_*, *R_VC_*, *I_VS_*, *I_VT1_*, *R_VT_*, *I_VT2_*, *R_VL_*, *I_VL_*, *S_F_*, *E_F_*, *I_F_*) in units of percent of the population, whereby the population of sand flies varies according to parameter *N_F_*. Axes labels and marginal distributions are placed in the main diagonal. Each point in a scatter plot represents the stationary solution of one simulation in 10000. Graphs are coloured according to the likelihood of the stationary solution: light green or grey for simulations which significantly differ from the maximum likelihood, and dark green for simulations which do not (according to a likelihood ratio test with 8 degrees of freedom). Parameters have been sampled from triangular distributions with modes given by the estimated mean and with upper and lower limits given by the 95% confidence interval for each parameter. Parameters have been sampled independently and are thus not correlated (see lines/columns 1 to 8). The coefficients of correlations in the table to the right have been computed from simulations of which the likelihood does not significantly differ from the maximum likelihood (green dots in the scatter plots). Main influences of the parameters are (see table to the right): {*N_F_*, *p_F2_*}∼*S_F_*, *f_HS_*∼{*I_HS_*, *I_HT1_*, *R_HT_*, *I_HT2_*, *R_HL_*, *I_HL_*}, *f_VS_*∼{*I_VS_*, *I_VT1_*, *R_VT_*, *I_VT2_*, *R_VL_*, *I_VL_*}, *η*∼{*S_V_*, *I_VP_*, *I_VD_*, *R_VD_*, *R_VC_*}, *γ_HD_*∼{*I_HD_*, *I_VD_*}, 1/*ρ_HD_*∼{*R_HD_*, *I_HP_* ; *R_VD_*}, 1/*ρ_HC_*∼{*S_H_*, *R_HC_*, *S_V_*, *R_VC_*}. For interrelationships between stationary solutions of the variables see Discussion in the main text.
(PPTX)

Table S1 **HIV parameters and variables.**
(DOC)

Text S1 **Supplemental text.**
(DOC)
