# Supplementary figures and images for: Visceral Leishmaniasis in the Indian Subcontinent: Modelling Epidemiology and Control
Source: PLoS Negl Trop Dis. 2011 Nov 29;5(11):e1405. doi: 10.1371/journal.pntd.0001405 (PMC3226461; doi:10.1371/journal.pntd.0001405)

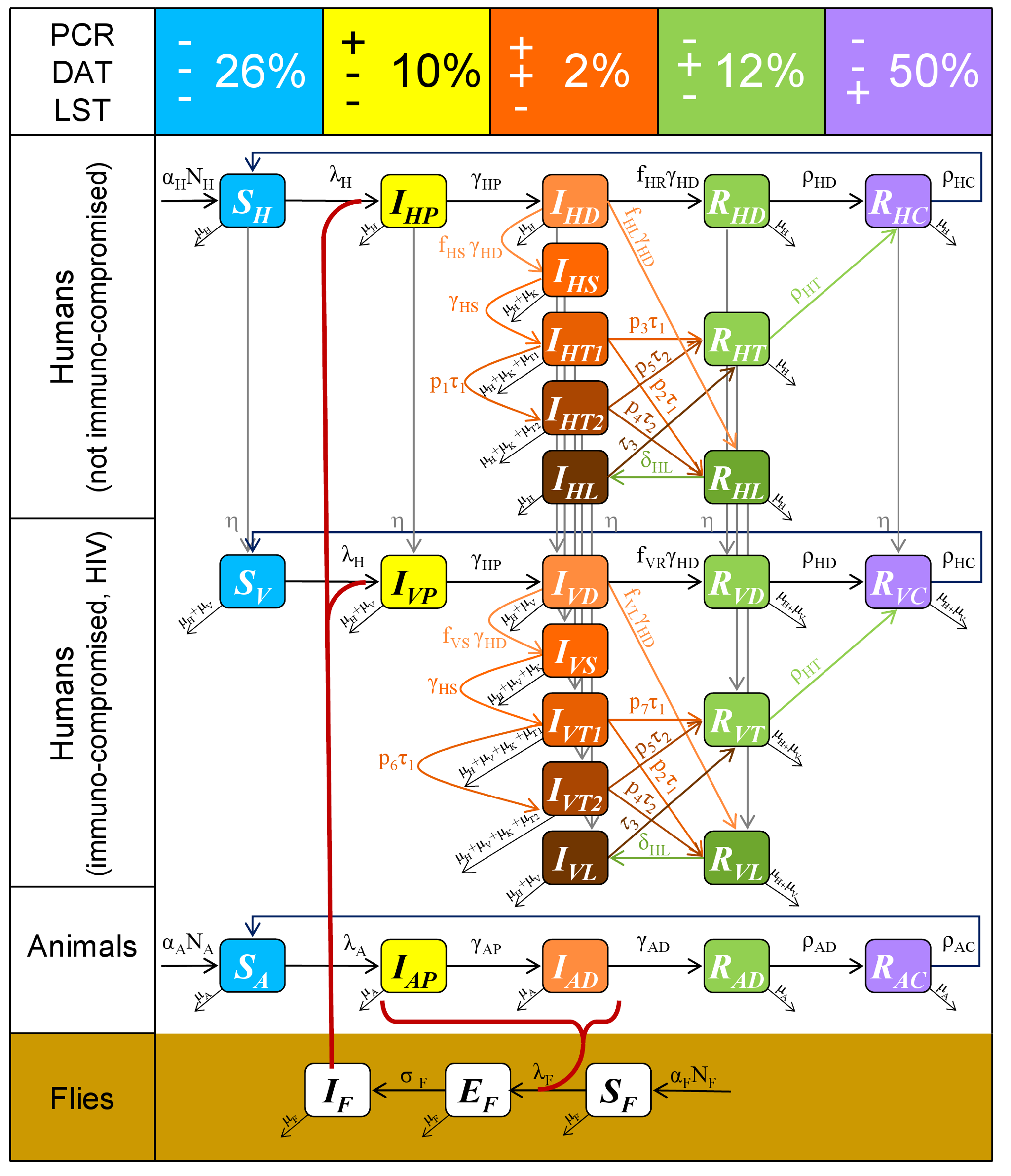

Supplement: Figure S1 — Full model. In addition to Fig. 1, this diagram shows the compartments of humans coinfected with HIV. HIV was modelled independently of infection with L. donovani and emerged with rate η from all human compartments. (TIF) [file pntd.0001405.s001.tif]
