# Supplementary material for: Visceral Leishmaniasis in the Indian Subcontinent: Modelling Epidemiology and Control
Source: PLoS Negl Trop Dis. 2011 Nov 29;5(11):e1405. doi: 10.1371/journal.pntd.0001405 (PMC3226461; doi:10.1371/journal.pntd.0001405)
Supplement: Figure S2 — Sensitivity analysis. Sensitivity analysis on the effects of parameter variations on the stationary solutions of the model. The scatter plot matrix lists in lines/columns 1 to 8 the eight estimated parameters (NF, pF2, fHS, fVS, η, 1/γHD, 1/ρHD, 1/ρHC), and in lines/columns 9 to 33 the stationary solutions of the model variables (SH, IHP, IHD, RHD, RHC, IHS, IHT1, RHT, IHT2, RHL, IHL, SV, IVP, IVD, RVD, RVC, IVS, IVT1, RVT, IVT2, RVL, IVL, SF, EF, IF) in units of percent of the population, whereby the population of sand flies varies according to parameter NF. Axes labels and marginal distributions are placed in the main diagonal. Each point in a scatter plot represents the stationary solution of one simulation in 10000. Graphs are coloured according to the likelihood of the stationary solution: light green or grey for simulations which significantly differ from the maximum likelihood, and dark green for simulations which do not (according to a likelihood ratio test with 8 degrees of freedom). Parameters have been sampled from triangular distributions with modes given by the estimated mean and with upper and lower limits given by the 95% confidence interval for each parameter. Parameters have been sampled independently and are thus not correlated (see lines/columns 1 to 8). The coefficients of correlations in the table to the right have been computed from simulations of which the likelihood does not significantly differ from the maximum likelihood (green dots in the scatter plots). Main influences of the parameters are (see table to the right): {NF, pF2}∼SF, fHS∼{IHS, IHT1, RHT, IHT2, RHL, IHL}, fVS∼{IVS, IVT1, RVT, IVT2, RVL, IVL}, η∼{SV, IVP, IVD, RVD, RVC}, γHD∼{IHD, IVD}, 1/ρHD∼{RHD, IHP ; RVD}, 1/ρHC∼{SH, RHC, SV, RVC}. For interrelationships between stationary solutions of the variables see Discussion in the main text. (PPTX) [file pntd.0001405.s002.pptx]

## Slide 1
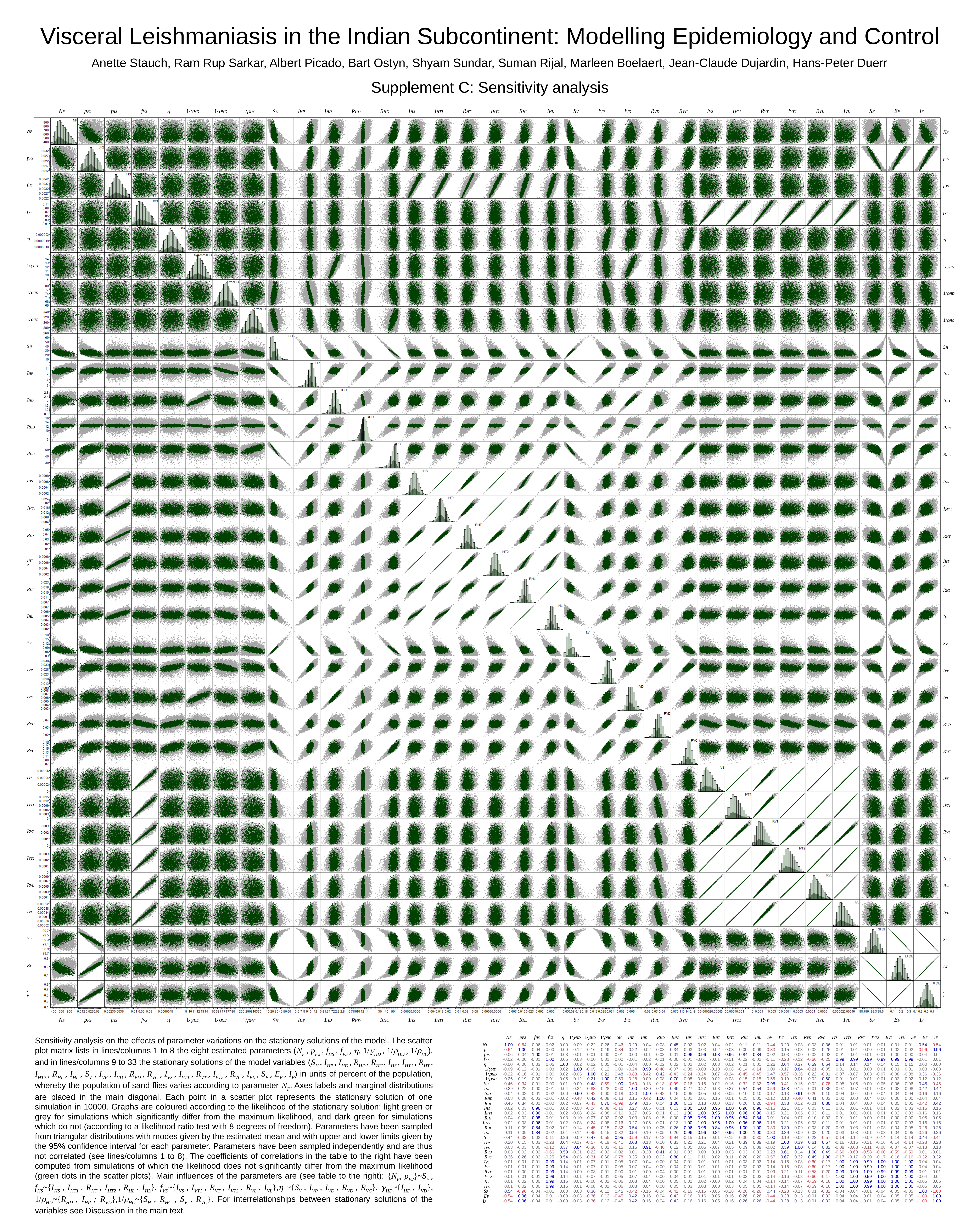

Visceral Leishmaniasis in the Indian Subcontinent: Modelling Epidemiology and Control
Anette Stauch, Ram Rup Sarkar, Albert Picado, Bart Ostyn, Shyam Sundar, Suman Rijal, Marleen Boelaert, Jean-Claude Dujardin, Hans-Peter Duerr
Supplement C: Sensitivity analysis
NF
pF2
fHS
fVS
1/gHD
1/rHD
IHP
IHD
RHC
IHS
IHT1
RHT
IHT2
RHL
IHL
SV
IVP
IVD
RVD
RVC
IVS
IVT1
RVT
IVT2
RVL
IVL
SF
EF
IF
h
1/rHC
SH
RHD
NF
pF2
fHS
fVS
h
1/gHD
1/rHD
1/rHC
SH
IHP
IHD
RHD
RHC
IHS
IHT1
RHT
IHT2
RHL
IHL
SV
IVP
IVD
RVD
RVC
IVS
IVT1
RVT
IVT2
RVL
IVL
SF
EF
IF
NF
pF2
fHS
fVS
h
1/gHD
1/rHD
1/rHC
SH
IHP
IHD
RHD
RHC
IHS
IHT1
RHT
IHT2
RHL
IHL
SV
IVP
IVD
RVD
RVC
IVS
IVT1
RVT
IVT2
RVL
IVL
SF
EF
IF
NF
pF2
fHS
fVS
1/gHD
1/rHD
IHP
IHD
RHC
IHS
IHT1
RHT
IHT2
RHL
IHL
SV
IVP
IVD
RVD
RVC
IVS
IVT1
RVT
IVT2
RVL
IVL
SF
EF
IF
h
1/rHC
SH
RHD
Sensitivity analysis on the effects of parameter variations on the stationary solutions of the model. The scatter plot matrix lists in lines/columns 1 to 8 the eight estimated parameters (NF , pF2 , fHS , fVS , h, 1/gHD , 1/rHD , 1/rHC), and in lines/columns 9 to 33 the stationary solutions of the model variables (SH , IHP , IHD , RHD , RHC , IHS , IHT1 , RHT , IHT2 , RHL , IHL , SV , IVP , IVD , RVD , RVC , IVS , IVT1 , RVT , IVT2 , RVL , IVL , SF , EF , IF) in units of percent of the population, whereby the population of sand flies varies according to parameter NF. Axes labels and marginal distributions are placed in the main diagonal. Each point in a scatter plot represents the stationary solution of one simulation in 10000. Graphs are coloured according to the likelihood of the stationary solution: light green or grey for simulations which significantly differ from the maximum likelihood, and dark green for simulations which do not (according to a likelihood ratio test with 8 degrees of freedom). Parameters have been sampled from triangular distributions with modes given by the estimated mean and with upper and lower limits given by the 95% confidence interval for each parameter. Parameters have been sampled independently and are thus not correlated (see lines/columns 1 to 8). The coefficients of correlations in the table to the right have been computed from simulations of which the likelihood does not significantly differ from the maximum likelihood (green dots in the scatter plots). Main influences of the parameters are (see table to the right): {NF, pF2}~SF , fHS~{IHS , IHT1 , RHT , IHT2 , RHL , IHL}, fVS~{IVS , IVT1 , RVT , IVT2 , RVL , IVL},h ~{SV , IVP , IVD , RVD , RVC}, gHD~{IHD , IVD}, 1/rHD~{RHD , IHP ; RVD},1/rHC~{SH , RHC , SV , RVC}. For interrelationships between stationary solutions of the variables see Discussion in the main text.
NF
pF2
fHS
fVS
h
1/gHD
1/rHD
1/rHC
SH
IHP
IHD
RHD
RHC
IHS
IHT1
RHT
IHT2
RHL
IHL
SV
IVP
IVD
RVD
RVC
IVS
IVT1
RVT
IVT2
RVL
IVL
SF
EF
IF
NF
pF2
fHS
fVS
h
1/gHD
1/rHD
1/rHC
SH
IHP
IHD
RHD
RHC
IHS
IHT1
RHT
IHT2
RHL
IHL
SV
IVP
IVD
RVD
RVC
IVS
IVT1
RVT
IVT2
RVL
IVL
SF
EF
IF
1.00
-0.64
-0.06
-0.02
-0.00
-0.09
-0.22
0.26
-0.46
0.29
0.04
0.08
0.45
0.02
0.02
-0.04
0.02
0.11
0.11
-0.44
0.20
0.03
0.03
0.36
0.01
0.01
-0.01
0.01
0.01
0.01
0.54
-0.54
-0.54
-0.64
1.00
-0.04
-0.00
-0.00
-0.12
-0.16
0.19
-0.34
0.22
-0.02
0.08
0.34
0.03
0.03
-0.02
0.03
0.09
0.09
-0.33
0.15
-0.03
0.02
0.26
0.01
0.01
-0.00
0.01
0.02
0.02
-0.96
0.96
0.96
-0.06
-0.04
1.00
-0.01
0.03
-0.01
-0.01
-0.00
0.01
0.00
-0.01
-0.03
-0.01
0.96
0.96
0.98
0.96
0.84
0.84
0.02
0.03
0.00
0.02
0.02
-0.01
-0.01
-0.01
-0.01
0.00
0.00
-0.04
0.04
0.04
-0.02
-0.00
-0.01
1.00
0.05
0.03
0.00
0.01
0.00
-0.01
0.02
-0.01
-0.00
-0.01
-0.01
-0.01
-0.01
-0.02
-0.02
-0.11
-0.28
-0.12
-0.66
-0.25
0.99
0.99
0.99
0.99
0.99
0.99
-0.01
0.01
0.01
-0.00
-0.00
0.03
0.05
1.00
0.02
0.02
0.05
-0.01
-0.04
0.00
-0.02
0.02
0.02
0.02
0.03
0.02
0.01
0.01
0.26
0.64
0.37
0.59
0.54
0.14
0.14
0.14
0.14
0.15
0.15
0.00
-0.00
-0.00
-0.09
-0.12
-0.01
0.03
0.02
1.00
-0.05
0.12
0.09
-0.24
0.90
-0.48
-0.07
-0.08
-0.08
-0.10
-0.08
-0.14
-0.14
0.09
-0.17
0.84
-0.21
-0.05
0.01
0.01
0.00
0.01
0.01
0.01
0.03
-0.03
-0.03
-0.22
-0.16
-0.01
0.00
0.02
-0.05
1.00
0.21
0.48
-0.83
-0.42
0.42
-0.43
-0.24
-0.24
0.07
-0.24
-0.45
-0.45
0.47
-0.57
-0.36
0.22
-0.31
-0.07
-0.07
0.03
-0.07
-0.08
-0.08
0.36
-0.36
-0.36
0.26
0.19
-0.00
0.01
0.05
0.12
0.21
1.00
-0.59
-0.28
-0.00
-0.08
0.70
-0.08
-0.08
-0.02
-0.08
-0.15
-0.15
-0.55
-0.19
0.01
-0.02
0.60
-0.01
-0.01
0.01
-0.01
-0.02
-0.02
-0.12
0.12
0.12
-0.46
-0.34
0.01
0.00
-0.01
0.09
0.48
-0.59
1.00
-0.60
-0.18
-0.13
-0.99
-0.16
-0.16
-0.02
-0.16
-0.32
-0.32
0.95
-0.41
-0.15
-0.02
-0.78
-0.05
-0.05
-0.00
-0.05
-0.06
-0.06
0.45
-0.45
-0.45
0.29
0.22
0.00
-0.01
-0.04
-0.24
-0.83
-0.28
-0.60
1.00
0.20
0.15
0.49
0.27
0.27
0.03
0.27
0.54
0.54
-0.59
0.68
0.15
0.01
0.35
0.07
0.07
-0.01
0.07
0.08
0.08
-0.42
0.42
0.42
0.04
-0.02
-0.01
0.02
0.00
0.90
-0.42
-0.00
-0.18
0.20
1.00
-0.42
0.15
0.05
0.05
-0.08
0.05
0.10
0.10
-0.17
0.13
0.91
-0.20
0.10
0.04
0.04
0.00
0.04
0.04
0.04
-0.16
0.16
0.16
0.08
0.08
-0.03
-0.01
-0.02
-0.48
0.42
-0.08
-0.13
0.15
-0.42
1.00
0.04
0.01
0.01
0.15
0.01
0.05
0.05
-0.12
0.10
-0.40
0.41
0.02
0.00
0.00
0.04
0.00
0.00
0.00
-0.04
0.04
0.04
0.45
0.34
-0.01
-0.00
0.02
-0.07
-0.43
0.70
-0.99
0.49
0.15
0.04
1.00
0.13
0.13
-0.00
0.13
0.26
0.26
-0.94
0.33
0.12
-0.01
0.80
0.04
0.04
0.00
0.04
0.05
0.05
-0.42
0.42
0.42
0.02
0.03
0.96
-0.01
0.02
-0.08
-0.24
-0.08
-0.16
0.27
0.05
0.01
0.13
1.00
1.00
0.95
1.00
0.96
0.96
-0.15
0.21
0.05
0.03
0.11
0.01
0.01
-0.01
0.01
0.02
0.03
-0.16
0.16
0.16
0.02
0.03
0.96
-0.01
0.02
-0.08
-0.24
-0.08
-0.16
0.27
0.05
0.01
0.13
1.00
1.00
0.95
1.00
0.96
0.96
-0.15
0.21
0.05
0.03
0.11
0.01
0.01
-0.01
0.01
0.02
0.03
-0.16
0.16
0.16
-0.04
-0.02
0.98
-0.01
0.03
-0.10
0.07
-0.02
-0.02
0.03
-0.08
0.15
-0.00
0.95
0.95
1.00
0.95
0.84
0.84
-0.01
0.04
-0.07
0.10
0.02
-0.01
-0.01
0.00
-0.01
-0.00
0.00
-0.05
0.05
0.05
0.02
0.03
0.96
-0.01
0.02
-0.08
-0.24
-0.08
-0.16
0.27
0.05
0.01
0.13
1.00
1.00
0.95
1.00
0.96
0.96
-0.15
0.21
0.05
0.03
0.11
0.01
0.01
-0.01
0.01
0.02
0.03
-0.16
0.16
0.16
0.11
0.09
0.84
-0.02
0.01
-0.14
-0.45
-0.15
-0.32
0.54
0.10
0.05
0.26
0.96
0.96
0.84
0.96
1.00
1.00
-0.30
0.39
0.09
0.03
0.20
0.03
0.03
-0.01
0.03
0.04
0.05
-0.26
0.26
0.26
0.11
0.09
0.84
-0.02
0.01
-0.14
-0.45
-0.15
-0.32
0.54
0.10
0.05
0.26
0.96
0.96
0.84
0.96
1.00
1.00
-0.30
0.39
0.09
0.03
0.20
0.03
0.03
-0.01
0.03
0.04
0.05
-0.26
0.26
0.26
-0.44
-0.33
0.02
-0.11
0.26
0.09
0.47
-0.55
0.95
-0.59
-0.17
-0.12
-0.94
-0.15
-0.15
-0.01
-0.15
-0.30
-0.30
1.00
-0.19
-0.02
0.23
-0.57
-0.14
-0.14
-0.09
-0.14
-0.14
-0.14
0.44
-0.44
-0.44
0.20
0.15
0.03
-0.28
0.64
-0.17
-0.57
-0.19
-0.41
0.68
0.13
0.10
0.33
0.21
0.21
0.04
0.21
0.39
0.39
-0.19
1.00
0.39
0.61
0.67
-0.16
-0.16
-0.21
-0.16
-0.14
-0.14
-0.28
0.28
0.28
0.03
-0.03
0.00
-0.12
0.37
0.84
-0.36
0.01
-0.15
0.15
0.91
-0.40
0.12
0.05
0.05
-0.07
0.05
0.09
0.09
-0.02
0.39
1.00
0.14
0.32
-0.08
-0.08
-0.11
-0.08
-0.07
-0.07
-0.13
0.13
0.13
0.03
0.02
0.02
-0.66
0.59
-0.21
0.22
-0.02
-0.02
0.01
-0.20
0.41
-0.01
0.03
0.03
0.10
0.03
0.03
0.03
0.23
0.61
0.14
1.00
0.49
-0.60
-0.60
-0.58
-0.60
-0.59
-0.59
0.01
-0.01
-0.01
0.36
0.26
0.02
-0.25
0.54
-0.05
-0.31
0.60
-0.78
0.35
0.10
0.02
0.80
0.11
0.11
0.02
0.11
0.20
0.20
-0.57
0.67
0.32
0.49
1.00
-0.17
-0.17
-0.20
-0.17
-0.16
-0.16
-0.32
0.32
0.32
0.01
0.01
-0.01
0.99
0.14
0.01
-0.07
-0.01
-0.05
0.07
0.04
0.00
0.04
0.01
0.01
-0.01
0.01
0.03
0.03
-0.14
-0.16
-0.08
-0.60
-0.17
1.00
1.00
0.99
1.00
1.00
1.00
-0.04
0.04
0.04
0.01
0.01
-0.01
0.99
0.14
0.01
-0.07
-0.01
-0.05
0.07
0.04
0.00
0.04
0.01
0.01
-0.01
0.01
0.03
0.03
-0.14
-0.16
-0.08
-0.60
-0.17
1.00
1.00
0.99
1.00
1.00
1.00
-0.04
0.04
0.04
-0.01
-0.00
-0.01
0.99
0.14
0.00
0.03
0.01
-0.00
-0.01
0.00
0.04
0.00
-0.01
-0.01
0.00
-0.01
-0.01
-0.01
-0.09
-0.21
-0.11
-0.58
-0.20
0.99
0.99
1.00
0.99
0.99
0.99
-0.01
0.01
0.01
0.01
0.01
-0.01
0.99
0.14
0.01
-0.07
-0.01
-0.05
0.07
0.04
0.00
0.04
0.01
0.01
-0.01
0.01
0.03
0.03
-0.14
-0.16
-0.08
-0.60
-0.17
1.00
1.00
0.99
1.00
1.00
1.00
-0.04
0.04
0.04
0.01
0.02
0.00
0.99
0.15
0.01
-0.08
-0.02
-0.06
0.08
0.04
0.00
0.05
0.02
0.02
-0.00
0.02
0.04
0.04
-0.14
-0.14
-0.07
-0.59
-0.16
1.00
1.00
0.99
1.00
1.00
1.00
-0.05
0.05
0.05
0.01
0.02
0.00
0.99
0.15
0.01
-0.08
-0.02
-0.06
0.08
0.04
0.00
0.05
0.03
0.03
0.00
0.03
0.05
0.05
-0.14
-0.14
-0.07
-0.59
-0.16
1.00
1.00
0.99
1.00
1.00
1.00
-0.05
0.05
0.05
0.54
-0.96
-0.04
-0.01
0.00
0.03
0.36
-0.12
0.45
-0.42
-0.16
-0.04
-0.42
-0.16
-0.16
-0.05
-0.16
-0.26
-0.26
0.44
-0.28
-0.13
0.01
-0.32
-0.04
-0.04
-0.01
-0.04
-0.05
-0.05
1.00
-1.00
-1.00
-0.54
0.96
0.04
0.01
-0.00
-0.03
-0.36
0.12
-0.45
0.42
0.16
0.04
0.42
0.16
0.16
0.05
0.16
0.26
0.26
-0.44
0.28
0.13
-0.01
0.32
0.04
0.04
0.01
0.04
0.05
0.05
-1.00
1.00
1.00
