# Supplementary material for: Visceral Leishmaniasis in the Indian Subcontinent: Modelling Epidemiology and Control
Source: PLoS Negl Trop Dis. 2011 Nov 29;5(11):e1405. doi: 10.1371/journal.pntd.0001405 (PMC3226461; doi:10.1371/journal.pntd.0001405)
Supplement: Table S1 — HIV parameters and variables. (DOC) [file pntd.0001405.s003.doc]

## Table S1 - HIV parameters and variables

For remaining values see Table 1, Table 2 and Table 3 in the main text.

|  | Description | Value | Reference |
| --- | --- | --- | --- |
| *μ_V_* | Excess mortality rate in HIV patients | 1/(5 years) | Assumed |
| *η* | HIV infection rate | 1.9 ∙ 10^-6^ day ^-1^ | Estimated, 95% CI (1.7 ∙ 10^-6^ to 2.1 ∙ 10^-6^) |
| *f_VS_* | Fraction of asymptomatically infected hosts (*I_VD_*) who will develop symptomatic KA | 0.04 | Estimated, 95% CI (0.0026 to 0.134) |
| *f_VL_* | Fraction of asymptomatically infected hosts (*I_VD_*) who will develop PKDL | 0.0001 | *= f_HL_* (assumed) |
| *f_VR_* | Fraction of asymptomatically infected hosts (*I_VD_*) who will recover without showing a symptomatic course of infection (→*R_VD_*) | 0.96 | = 1 – (*f_VS_* + *f_VL_*) |
| *P_V_* | Prevalence of HIV | 0.3% | National AIDS Control Organisation (NACO) |
| *p_F5_* | Probability that a susceptible fly becomes infected when feeding on a human host of types *I_VS_*, *I_VT1_*, *I_VT2_* | = *p_F3_*= 1 | Assumed |
| *p_F6_* | Probability that a susceptible fly becomes infected when feeding on a human host of type *I_VL_* | = *p_F4_*= 1 | Assumed |
| *p_6_* | Fraction of immuno-compromised KA patients not responding to KA first-line treatment (conditional on surviving treatment, 1-*f_T_*) | 30% (100% = *p_6_* + *p_7_* +*p_2_*) | [14] |
| *P_7_* | Fraction of immuno-compromised KA patients recovering during KA first-line treatment (conditional on surviving treatment, 1-*f_T_*) | 67% (100% = *p_6_*+*p_7_*+ *p_2_*) | =1 - (*p_6_* + *p_2_*) |
