# Supplementary material for: Visceral Leishmaniasis in the Indian Subcontinent: Modelling Epidemiology and Control
Source: PLoS Negl Trop Dis. 2011 Nov 29;5(11):e1405. doi: 10.1371/journal.pntd.0001405 (PMC3226461; doi:10.1371/journal.pntd.0001405)
Supplement: Text S1 — Supplemental text. (DOC) [file pntd.0001405.s004.doc]

## Supplement

## Supplement A: Description of the full model

The transmission dynamics of *L. donovani* in the Indian subcontinent was modelled deterministically by a system of ordinary differential equations. Figure S1 describes the model graphically. Table 1, Table 2 and Table 3 in the main text provide lists of parameters and variables except those on immuno-compromised patients, which are listed in Table S1. We used three diagnostic markers to categorise the people living in endemic areas: PCR (index P, earliest marker for *L. donovani* infection), DAT (index D, antibody response), and LST (index C, suggesting a state of 'cellular' immunity). Superscript "+" and "-" represent positive and negative results. The index X is used in the following as a placeholder for the type of host: index H for humans, index V for immuno-compromised humans and index A for animals. If the infection proceeded asymptomatically, we assumed that a host typically passed through the following five stages:

***S_X_***: *Susceptible stage* (**P^-^D^-^C^-^**). Hosts are negative for all three markers and can become infected in the future.

***I_XP_***: *Early asymptomatic, infectious stage* (**P^+^D^-^C^-^**). The parasite can be detected by PCR, but there is not yet any humoral or cellular response in the host.

***I_XD_***: *Late asymptomatic, infectious stage* (**P^+^D^+^C^-^**). Hosts are still PCR-positive, and antibodies can be detected by DAT.

***R_XD_***: *Early recovered stage* (**P^-^D^+^C^-^**). The parasite cannot be detected anymore, but hosts are DAT-positive and not yet LST-positive.

***R_XC_***: *Late recovered stage* (**P^-^D^-^C^+^**). Hosts are DAT-negative but still LST-positive and assumed to be protected against re-infection.

The model used constant population sizes based on constant birth and death rates. Notations are based on the following rules: Indices of variables and parameters consist of a capital letter at position one referring to host type (*H*, *A*, or *F*) and a second letter at position two referring to the diagnostic state (*P*, *D*, or *C*). Additional indices represent disease and treatment status in human hosts as follows: *S*, Symptomatic disease; *T*, treatment; *T1*, first-line treatment; *T2*, second-line treatment; and *L*, PKDL. Greek letters denote rates (birth rate *α*, death rate *μ*, biting rate *β*, infection rate *λ*, recovery rate *γ,* loss rate *ρ, *treatment rate *τ*, relapse rate *δ*); *f* and *p* denote fractions and proportions or probabilities, respectively; and transitions are indicated by an arrow (→).

### Natural history of infection in humans

Humans are born as susceptibles (*S_H_*) at a per capita birth rate *α_H_*. We assume a constant population size based on a constant birth and death rate (*μ_H_*). Susceptibles are infected at rate *λ_H_*, that depends on 1) the biting rate *β* of sand flies, 2) the anthropophilic factor *a*, which indicates the sand flies' preference for animal or human hosts, 3) the probability of becoming infected when being bitten by an infectious sand fly (*p_H_*), and 4) the number of infectious sand flies per host.

After infection, humans enter the early asymptomatic stage *I_HP_* and become PCR-positive in peripheral blood. Seroconversion occurs at rate *γ_HP_*, leading to the late asymptomatic stage *I_HD_*, which is characterised by onset of DAT-positivity. If not dying, humans remain in this asymptomatic stage for 1/*γ_HD_* days. Infection with leishmaniasis proceeds asymptomatically in most cases, with only a minor fraction of cases develops KA. A major fraction *f_HR_* of these cases does not develop disease, becomes PCR-negative in the early-recovered stage *R_HD_*, and develops LST-positivity (*R_HC_*) at rate *ρ_HD_*. A remaining fraction of 1‑*f_HR_* asymptomatic infections is split into a tiny fraction *f_HL_* of putatively recovering humans who develop a state of PCR-negativity in peripheral blood, while still harboring a non-detectable number of parasites. This stage is denoted as *R_HL_*, from where relapse to PKDL (*I_HL_*) follows. A fraction *f_HS_* develops symptomatic KA (*I_HS_*) while maintaining PCR-positive.

### Therapy

The fraction *f_HS_* developing symptomatic KA (*I_HS_*) is eligible for treatment. If not dying, these patients receive first-line treatment (*I_HT1_*) on average after 1/*γ_HS_* days. First-line treatment (in state *I_HT1_*) is given for 1/*τ_1_* days. A proportion *p_3_* of patients clears parasites under first-line treatment, recovers (→*R_HT_*), and finally becomes LST-positive (→*R_HC_*) as in asymptomatic infections. The remaining proportion 1-*p_3_* of patients represents treatment failures; these are split into PCR-positive proportion *p_1_* (KA patients receiving second-line treatment, *I_HT2_*) and into a proportion *p_2_* of patients putatively recovering into a state of false PCR-negativity while still harboring a non-detectable number of parasites (*R_HL_*, from where relapse to PKDL, *I_HL_*, will follow).

Second-line treatment (in state *I_HT2_*) is given for 1/*τ_2_* days. As for first-line treatment, a proportion *p_5_* of patients under second-line treatment recovers (→*R_HT_*), and becomes LST-positive (→*R_HC_*). The remaining proportion of *p_4_* patients putatively recovers into a state of PCR-negativity (*R_HL_*) from which, again, relapse to PCR-positivity and PKDL (*I_HL_*) follows for those who did not die on average after 1/*δ_HL_* days. All PKDL patients (*I_HL_*) are treated until full recovery (→*R_HT_*→*R_HC_*).

Excess mortality 1) affects KA patients whose mortality rate increases the baseline mortality rate *μ_H_* to *μ__* *=* *μ_H_* *+* *μ_K_*, and 2) occurs under treatment because of drug toxicity. Five percent of patients die because of first-line or second-line treatment; here the mortality rate increases to *μ_2_* *=* *μ_H_* *+* *μ_K_* *+* *μ_T1_* and *μ_3_* *=* *μ_H_* *+* *μ_K_* *+* *μ_T2_*. LST-positivity (suggesting a state of cellular immunity) may remain over a period of 1/*ρ_HC_* days, after which recovered humans (*R_HC_*) again become negative for all diagnostic markers (→*S_H_*).

### Animals

Animals can either act as infection sinks or reservoirs; their role in the transmission cycle of *L. donovani* in the Indian subcontinent, however, is unclear and was neglected for model calibration and parameter estimation. Animal hosts are represented in Fig. S1 for purposes of completeness, with a natural history of infection similar to that in humans but without any symptomatic stage of disease. In this investigation, we set the anthropophilic factor *a*= 1, i.e., flies feed exclusively on human hosts and not on animals.

### Vectors

We considered sand flies in the susceptible (*S_F_*), latent (*E_F_*, not infectious), or infectious (*I_F_*) stages. Flies can become infected by blood meals taken on hosts of all infectious states (*I_HX_*, *I_VX_*, *I_AX_*), whereby index X is a placeholder for the different disease states (*P*, *D*, *S*, *T1*, *T2*, *L*). The infection rate *λ_F_* of flies is determined by 1) their biting rate *β*, 2) the anthropophilic factor *a* (not considered in this investigation by setting *a* = 1), 3) the infection probabilities of flies dependent on the infection status of the hosts (*p_F1_* to *p_F4_*, see Table 2), and 4) the number of infectious hosts.

### Immuno-compromised humans

We assumed that infection with HIV occur independent of infection with VL and thus emerges with rate *η* from all human compartments. However, the proportion of patients with symptomatic disease is higher for patients coinfected with HIV (see parameter *f_VS_* instead of *f_HS_* in Table S1) and they have a higher fraction of KA patients not responding to KA first-line treatment (see parameter *p_6_* instead of *p_1_* in Table S1). The life expectancy of HIV patients is reduced for all these compartments because of the excess mortality rate *μ_V_*.

### Equations

| Humans | $\begin{matrix} \frac{dS_{H}}{dt}=\alpha_{H}N_{H}+\rho_{HC}R_{HC}-\left(\lambda_{H}+\mu_{H}+\eta\right)S_{H} \\ \frac{dI_{HP}}{dt}=\lambda_{H}S_{H}-\left(\gamma_{HP}+\mu_{H}+\eta\right)I_{HP} \\ \frac{dI_{HD}}{dt}=\gamma_{HP}I_{HP}-\left(\gamma_{HD}+\mu_{H}+\eta\right)I_{HD} \\ \frac{dR_{HD}}{dt}=f_{HR}\gamma_{HD}I_{HD}-\left(\rho_{HD}+\mu_{H}+\eta\right)R_{HD} \\ \frac{dR_{HC}}{dt}=\rho_{HD}R_{HD}+\rho_{HT}R_{HT}-\left(\rho_{HC}+\mu_{H}+\eta\right)R_{HC} \\ \frac{dI_{HS}}{dt}=f_{HS}\gamma_{HD}I_{HD}-\left(\gamma_{HS}+\mu_{H}+\mu_{K}+\eta\right)I_{HS} \\ \frac{dI_{HT1}}{dt}=\gamma_{HS}I_{HS}-\left(\left(1-f_{T}\right)\tau_{1}+\mu_{H}+\mu_{K}+\mu_{T1}+\eta\right)I_{HT1} \\ \frac{dR_{HT}}{dt}=p_{3}\tau_{1}I_{HT1}+p_{5}\tau_{2}I_{HT2}+\tau_{3}I_{HL}-\left(\rho_{HT}+\mu_{H}+\eta\right)R_{HT} \\ \frac{dI_{HT2}}{dt}=p_{1}\tau_{1}I_{HT1}-\left(\left(1-f_{T}\right)\tau_{2}+\mu_{H}+\mu_{K}+\mu_{T2}+\eta\right)I_{HT2} \\ \frac{dR_{HL}}{dt}=p_{2}\tau_{1}I_{HT1}+p_{4}\tau_{2}I_{HT2}+f_{HL}\gamma_{HD}I_{HD}-\left(\delta_{HL}+\mu_{H}+\eta\right)R_{HL} \\ \frac{dI_{HL}}{dt}=\delta_{HL}R_{HL}-\left(\tau_{3}+\mu_{H}+\eta\right)I_{HL} \end{matrix}$ whereby $\mu_{T1}=f_{T}\cdot\tau_{1}$ and $\mu_{T2}=f_{T}\cdot\tau_{2}$ |
| --- | --- |
| Humans coinfected with HIV Extra mortality due to coinfection with HIV (*μ_V_*), symptomatic KA (*μ_K_*), or treatment (*μ_T1_*, *μ_T2_*) was compensated for the birth rate *α* such that a constant population size was maintained. | $\begin{matrix} \frac{dS_{V}}{dt}=\eta S_{H}+\rho_{HC}R_{VC}-\left(\lambda_{H}+\mu_{H}+\mu_{V}\right)S_{H} \\ \frac{dI_{VP}}{dt}=\eta I_{HP}+\lambda_{H}S_{V}-\left(\gamma_{HP}+\mu_{H}+\mu_{V}\right)I_{VP} \\ \frac{dI_{VD}}{dt}=\eta I_{HD}+\gamma_{HP}I_{VP}-\left(\gamma_{HD}+\mu_{H}+\mu_{V}\right)I_{VD} \\ \frac{dR_{VD}}{dt}=\eta R_{HD}+f_{VR}\gamma_{HD}I_{VD}-\left(\rho_{HD}+\mu_{H}+\mu_{V}\right)R_{VD} \\ \frac{dR_{VC}}{dt}=\eta R_{HC}+\rho_{HD}R_{VD}+\rho_{HT}R_{VT}-\left(\rho_{HC}+\mu_{H}+\mu_{V}\right)R_{VC} \\ \frac{dI_{VS}}{dt}=\eta I_{HS}+f_{VS}\gamma_{HD}I_{VD}-\left(\gamma_{HS}+\mu_{H}+\mu_{K}+\mu_{V}\right)I_{VS} \\ \frac{dI_{VT1}}{dt}=\eta I_{HT1}+\gamma_{HS}I_{VS}-\left(\left(1-f_{T}\right)\tau_{1}+\mu_{H}+\mu_{K}+\mu_{V}+\mu_{T1}\right)I_{VT1} \\ \frac{dR_{VT}}{dt}=\eta R_{HT}+p_{7}\tau_{1}I_{VT1}+p_{5}\tau_{2}I_{VT2}+\tau_{3}I_{VL}-\left(\rho_{HT}+\mu_{H}+\mu_{V}\right)R_{VT} \\ \frac{dI_{VT2}}{dt}=\eta I_{HT2}+p_{6}\tau_{1}I_{VT1}-\left(\left(1-f_{T}\right)\tau_{2}+\mu_{H}+\mu_{K}+\mu_{V}+\mu_{T2}\right)I_{VT2} \\ \frac{dR_{VL}}{dt}=\eta R_{HL}+p_{2}\tau_{1}I_{VT1}+p_{4}\tau_{2}I_{VT2}+f_{VL}\gamma_{HD}I_{VD}-\left(\delta_{HL}+\mu_{H}+\mu_{V}\right)R_{VL} \\ \frac{dI_{VL}}{dt}=\eta I_{HL}+\delta_{HL}R_{VL}-\left(\tau_{3}+\mu_{H}+\mu_{V}\right)I_{VL} \end{matrix}$ |
| Animals | $\begin{matrix} \frac{dS_{A}}{dt}=\alpha_{A}N_{A}+\rho_{AC}R_{AC}-\left(\lambda_{A}+\mu_{A}\right)S_{A} \\ \frac{dI_{AP}}{dt}=\lambda_{A}S_{A}-\left(\gamma_{AP}+\mu_{A}\right)I_{AP} \\ \frac{dI_{AD}}{dt}=\gamma_{AP}I_{AP}-\left(\gamma_{AD}+\mu_{A}\right)I_{AD} \\ \frac{dR_{AD}}{dt}=\gamma_{AD}I_{AD}-\left(\rho_{AD}+\mu_{A}\right)R_{AD} \\ \frac{dR_{AC}}{dt}=\rho_{AD}R_{AD}-\left(\rho_{AC}+\mu_{A}\right)R_{AC} \end{matrix}$ |
| Flies | $\begin{matrix} \frac{dS_{F}}{dt}=\alpha_{F}N_{F}-\left(\lambda_{F}+\mu_{F}\right)S_{F} \\ \frac{dE_{F}}{dt}=\lambda_{F}S_{F}-\left(\sigma_{F}+\mu_{F}\right)E_{F} \\ \frac{dI_{F}}{dt}=\sigma_{F}E_{F}-\mu_{F}I_{F} \end{matrix}$ |
| Infection rate *λ* | $\begin{matrix} \lambda_{H}=\frac{\beta\cdot a\cdot p_{H}\cdot I_{F}}{N_{H}\cdot a+N_{A}\left(1-a\right)} \\ \lambda_{A}=\frac{\beta\left(1-a\right)\cdot p_{A}\cdot I_{F}}{N_{H}\cdot a+N_{A}\left(1-a\right)} \\ \lambda_{F}=\frac{\beta\left(a\cdot I_{H}+\left(1-a\right)I_{A}\right)}{N_{H}\cdot a+N_{A}\left(1-a\right)} \end{matrix}$ |
| The equation $I_{H}=p_{F1}\left(I_{HP}+I_{VP}\right)+p_{F2}\left(I_{HD}+I_{VD}\right)+p_{F3}\left(I_{HS}+I_{HT1}+I_{HT2}\right)+p_{F4}I_{HL}+p_{F5}\left(I_{VS}+I_{VT1}+I_{VT2}\right)+p_{F6}I_{VL}$ denotes the proportion of infected hosts among the human population, and $I_{A}=p_{FA}\left(p_{F1}\cdot I_{AP}+p_{F2}\cdot I_{AD}\right)$ is the proportion of infected hosts among the animal population. | |

## Supplement B: Calculation of the basic reproduction number *R_0_*

*R_0_* is calculated from the next generation matrix [48] considering the four combinations of infections between humans who are HIV-positive or -negative, with elements as follows: *a*: infections of HIV-negative humans via flies infected by HIV-negative humans, *b*: HIV-positive humans infected via flies infected by HIV-negative humans, *c*: HIV-negative humans infected via flies infected by HIV-positive humans, *d*: infections of HIV- positive humans via flies infected by HIV- positive humans. With these elements the next generation matrix is given by

$M=\left(\begin{matrix} a & b \\ c & d \end{matrix}\right)$.

For purposes of comprehensive mathematical notation we use the following abbreviations:

*D*'s denote mean sojourn times in infectious compartments as denoted by the corresponding indices, and *f* 's denote the fractions of infected humans who undergo transition to the downstream compartment.

For humans not infected with HIV these are:

$D_{HP}=\frac{1}{\gamma_{HP}+\mu_{H}+\eta}$, $D_{HD}=\frac{1}{\gamma_{HD}+\mu_{H}+\eta}$, $D_{HS}=\frac{1}{\gamma_{HS}+\mu_{H}+\mu_{K}+\eta}$, $D_{HT1}=\frac{1}{\tau_{1}+\mu_{H}+\mu_{K}+\eta}$, $D_{HT2}=\frac{1}{\tau_{2}+\mu_{H}+\mu_{K}+\eta}$, $D_{RHL}=\frac{1}{\delta_{HL}+\mu_{H}+\eta}$ and $D_{HL}=\frac{1}{\tau_{3}+\mu_{H}+\eta}$.

$f_{HP}=\frac{\gamma_{HP}}{\gamma_{HP}+\mu_{H}+\eta}$, $f_{HD}=\frac{f_{HS}\cdot\gamma_{HD}}{\gamma_{HD}+\mu_{H}+\eta}$, $f_{HD2}=\frac{f_{HL}\cdot\gamma_{HD}}{\gamma_{HD}+\mu_{H}+\eta}$, $f_{H\_S}=\frac{\gamma_{HS}}{\gamma_{HS}+\mu_{H}+\mu_{K}+\eta}$, $f_{HT1}=\frac{p_{1}\cdot\tau_{1}}{\tau_{1}+\mu_{H}+\mu_{K}+\eta}$, $f_{HT12}=\frac{p_{2}\cdot\tau_{2}}{\tau_{2}+\mu_{H}+\mu_{K}+\eta}$, $f_{HT2}=\frac{p_{4}\cdot\tau_{2}}{\tau_{2}+\mu_{H}+\mu_{K}+\eta}$ and $f_{RHL}=\frac{\delta_{HL}}{\delta_{HL}+\mu_{H}+\eta}$.

For humans infected with HIV these are:

$D_{VP}=\frac{1}{\gamma_{HP}+\mu_{H}+\mu_{V}}$, $D_{VD}=\frac{1}{\gamma_{HD}+\mu_{H}+\mu_{V}}$, $D_{VS}=\frac{1}{\gamma_{HS}+\mu_{H}+\mu_{K}+\mu_{V}}$, $D_{VT1}=\frac{1}{\tau_{1}+\mu_{H}+\mu_{K}+\mu_{V}}$, $D_{VT2}=\frac{1}{\tau_{2}+\mu_{H}+\mu_{K}+\mu_{V}}$, $D_{RVL}=\frac{1}{\delta_{HL}+\mu_{H}+\mu_{V}}$ and $D_{VL}=\frac{1}{\tau_{3}+\mu_{H}+\mu_{V}}$.

$f_{VP}=\frac{\gamma_{HP}}{\gamma_{HP}+\mu_{H}+\mu_{V}}$, $f_{VD}=\frac{f_{VS}\cdot\gamma_{HD}}{\gamma_{HD}+\mu_{H}+\mu_{V}}$, $f_{VD2}=\frac{f_{VL}\cdot\gamma_{HD}}{\gamma_{HD}+\mu_{H}+\eta}$, $f_{VS}=\frac{\gamma_{HS}}{\gamma_{HS}+\mu_{H}+\mu_{K}+\mu_{V}}$, $f_{VT1}=\frac{p_{6}\cdot\tau_{1}}{\tau_{1}+\mu_{H}+\mu_{K}+\mu_{V}}$, $f_{VT12}=\frac{p_{2}\cdot\tau_{2}}{\tau_{2}+\mu_{H}+\mu_{K}+\eta}$, $f_{VT2}=\frac{p_{4}\cdot\tau_{2}}{\tau_{2}+\mu_{H}+\mu_{K}+\mu_{V}}$ and $f_{RVL}=\frac{\delta_{HL}}{\delta_{HL}+\mu_{H}+\mu_{V}}$.

In the following equations, *s* denotes the sand fly part which is similar for all compartments and which is given by $s=\left(\frac{\beta\cdot a}{N_{H}\cdot a+N_{A}\cdot\left(1-a\right)}\right)^{2}\cdot p_{H}\cdot N_{F}\cdot\frac{\sigma_{F}}{\sigma_{F}+\mu_{F}}\cdot\frac{1}{\mu_{F}}\cdot N_{H}$. It reduces in the current model without animals (*a* = 1) to $s=\left(\frac{\beta}{N_{H}}\right)^{2}\cdot p_{H}\cdot N_{F}\cdot\frac{\sigma_{F}}{\sigma_{F}+\mu_{F}}\cdot\frac{1}{\mu_{F}}\cdot N_{H}$. Furthermore, the prevalence of HIV in the study area is *P_V_*= 0.003 (see Table S1).

With these notations element *a* in matrix *M* is given by

$$a=R_{IHP\_1}+R_{IHD\_1}+R_{IHS\_1}+R_{IHT1\_1}+R_{IHT2\_1}+R_{IHL\_1}$$

whereby

$$\begin{matrix} R_{IHP\_1}=\left(D_{HP}\cdot p_{F1}+\eta\cdot D_{HP}\left(D_{VP}\cdot p_{F1}+f_{VP}\left(D_{VD}\cdot p_{F2}+f_{VD}\left(\begin{matrix} D_{VS}\cdot p_{F3}+f_{VS} \\ \left(\begin{matrix} D_{VT1}\cdot p_{F3}+f_{VT1} \\ \left(\begin{matrix} D_{VT2}\cdot p_{F3} \\ +f_{VT2}\cdot f_{RVL}\cdot D_{VL}\cdot p_{F4} \end{matrix}\right) \end{matrix}\right) \end{matrix}\right)\right)\right)\right)s\left(1-P_{V}\right) \\ +\eta\cdot D_{HP}\cdot f_{VP}\cdot f_{VD2}\cdot f_{RVL}\cdot D_{VL}\cdot p_{F4}\cdot s\left(1-P_{V}\right) \\ +\eta\cdot D_{HP}\cdot f_{VP}\cdot f_{VD}\cdot f_{VS}\cdot f_{VT12}\cdot f_{RVL}\cdot D_{VL}\cdot p_{F4}\cdot s\left(1-P_{V}\right) \end{matrix}$$

$$\begin{matrix} R_{IHD\_1}=f_{HP}\left(D_{HD}\cdot p_{F2}+\eta\cdot D_{HD}\left(D_{VD}\cdot p_{F2}+f_{VD}\left(D_{VS}\cdot p_{F3}+f_{VS}\left(\begin{matrix} D_{VT1}\cdot p_{F3}+f_{VT1} \\ \left(\begin{matrix} D_{VT2}\cdot p_{F3} \\ +f_{VT2}\cdot f_{RVL}\cdot D_{VL}\cdot p_{F4} \end{matrix}\right) \end{matrix}\right)\right)\right)\right)s\left(1-P_{V}\right) \\ +\eta\cdot D_{HD}\cdot f_{VD2}\cdot f_{RVL}\cdot D_{VL}\cdot p_{F4}\cdot s\left(1-P_{V}\right) \\ +\eta\cdot D_{HD}\cdot f_{VD}\cdot f_{VS}\cdot f_{VT12}\cdot f_{RVL}\cdot D_{VL}\cdot p_{F4}\cdot s\left(1-P_{V}\right) \end{matrix}$$

$$\begin{matrix} R_{IHS\_1}=f_{HP}\cdot f_{HD}\left(D_{HS}\cdot p_{F3}+\eta\cdot D_{HS}\left(D_{VS}\cdot p_{F3}+f_{VS}\left(D_{VT1}\cdot p_{F3}+f_{VT1}\left(\begin{matrix} D_{VT2}\cdot p_{F3} \\ +f_{VT2}\cdot f_{RVL}\cdot D_{VL}\cdot p_{F4} \end{matrix}\right)\right)\right)\right)s\left(1-P_{V}\right) \\ +\eta\cdot D_{HS}\cdot f_{VS}\cdot f_{VT12}\cdot f_{RVL}\cdot D_{VL}\cdot p_{F4}\cdot s\left(1-P_{V}\right) \end{matrix}$$

$$\begin{matrix} R_{IHT1\_1}=f_{HP}\cdot f_{HD}\cdot f_{H\_S}\left(D_{HT1}\cdot p_{F3}+\eta\cdot D_{HT1}\left(D_{VT1}\cdot p_{F3}+f_{VT1}\left(D_{VT2}\cdot p_{F3}+f_{VT2}\cdot f_{RVL}\cdot D_{VL}\cdot p_{F4}\right)\right)\right)s\left(1-P_{V}\right) \\ +\eta\cdot D_{HT1}\cdot f_{VT12}\cdot f_{RVL}\cdot D_{VL}\cdot p_{F4}\cdot s\left(1-P_{V}\right) \end{matrix}$$

$$R_{IHT2\_1}=f_{HP}\cdot f_{HD}\cdot f_{H\_S}\cdot f_{HT1}\left(D_{HT2}\cdot p_{F3}+\eta\cdot D_{HT2}\left(D_{VT2}\cdot p_{F3}+f_{VT2}\cdot f_{RVL}\cdot D_{VL}\cdot p_{F4}\right)\right)s\left(1-P_{V}\right)$$

$$\begin{matrix} R_{IHL\_1}=f_{HP}\cdot f_{HD}\cdot f_{H\_S}\cdot f_{HT1}\cdot f_{HT2}\left(f_{RHL}\left(D_{HL}+\eta\cdot D_{HL}\cdot D_{VL}\right)+\eta\cdot D_{RHL}\cdot f_{RVL}\cdot D_{VL}\right)p_{F4}\cdot s\left(1-P_{V}\right) \\ +f_{HP}\cdot f_{HD2}\left(f_{RHL}\left(D_{HL}+\eta\cdot D_{HL}\cdot D_{VL}\right)+\eta\cdot D_{RHL}\cdot f_{RVL}\cdot D_{VL}\right)p_{F4}\cdot s\left(1-P_{V}\right) \\ +f_{HP}\cdot f_{HD}\cdot f_{H\_S}\cdot f_{HT12}\left(f_{RHL}\left(D_{HL}+\eta\cdot D_{HL}\cdot D_{VL}\right)+\eta\cdot D_{RHL}\cdot f_{RVL}\cdot D_{VL}\right)p_{F4}\cdot s\left(1-P_{V}\right) \end{matrix}$$

Element *b* in matrix *M* is given by

$$b=R_{IHP\_2}+R_{IHD\_2}+R_{IHS\_2}+R_{IHT1\_2}+R_{IHT2\_2}+R_{IHL\_2}$$

whereby

$$\begin{matrix} R_{IHP\_2}=\left(D_{HP}\cdot p_{F1}+\eta\cdot D_{HP}\left(D_{VP}\cdot p_{F1}+f_{VP}\left(D_{VD}\cdot p_{F2}+f_{VD}\left(\begin{matrix} D_{VS}\cdot p_{F3}+f_{VS} \\ \left(\begin{matrix} D_{VT1}\cdot p_{F3}+f_{VT1} \\ \left(\begin{matrix} D_{VT2}\cdot p_{F3} \\ +f_{VT2}\cdot f_{RVL}\cdot D_{VL}\cdot p_{F4} \end{matrix}\right) \end{matrix}\right) \end{matrix}\right)\right)\right)\right)s\cdot P_{V} \\ +\eta\cdot D_{HP}\cdot f_{VP}\cdot f_{VD2}\cdot f_{RVL}\cdot D_{VL}\cdot p_{F4}\cdot s\cdot P_{V} \\ +\eta\cdot D_{HP}\cdot f_{VP}\cdot f_{VD}\cdot f_{VS}\cdot f_{VT12}\cdot f_{RVL}\cdot D_{VL}\cdot p_{F4}\cdot s\cdot P_{V} \end{matrix}$$

$$\begin{matrix} R_{IHD\_2}=f_{HP}\left(D_{HD}\cdot p_{F2}+\eta\cdot D_{HD}\left(D_{VD}\cdot p_{F2}+f_{VD}\left(D_{VS}\cdot p_{F3}+f_{VS}\left(\begin{matrix} D_{VT1}\cdot p_{F3}+f_{VT1} \\ \left(\begin{matrix} D_{VT2}\cdot p_{F3} \\ +f_{VT2}\cdot f_{RVL}\cdot D_{VL}\cdot p_{F4} \end{matrix}\right) \end{matrix}\right)\right)\right)\right)s\cdot P_{V} \\ +\eta\cdot D_{HD}\cdot f_{VD2}\cdot f_{RVL}\cdot D_{VL}\cdot p_{F4}\cdot s\cdot P_{V} \\ +\eta\cdot D_{HD}\cdot f_{VD}\cdot f_{VS}\cdot f_{VT12}\cdot f_{RVL}\cdot D_{VL}\cdot p_{F4}\cdot s\cdot P_{V} \end{matrix}$$

$$\begin{matrix} R_{IHS\_2}=f_{HP}\cdot f_{HD}\left(D_{HS}\cdot p_{F3}+\eta\cdot D_{HS}\left(D_{VS}\cdot p_{F3}+f_{VS}\left(D_{VT1}\cdot p_{F3}+f_{VT1}\left(\begin{matrix} D_{VT2}\cdot p_{F3} \\ +f_{VT2}\cdot f_{RVL}\cdot D_{VL}\cdot p_{F4} \end{matrix}\right)\right)\right)\right)s\cdot P_{V} \\ +\eta\cdot D_{HS}\cdot f_{VS}\cdot f_{VT12}\cdot f_{RVL}\cdot D_{VL}\cdot p_{F4}\cdot s\cdot P_{V} \end{matrix}$$

$$\begin{matrix} R_{IHT1\_2}=f_{HP}\cdot f_{HD}\cdot f_{H\_S}\left(D_{HT1}\cdot p_{F3}+\eta\cdot D_{HT1}\left(D_{VT1}\cdot p_{F3}+f_{VT1}\left(D_{VT2}\cdot p_{F3}+f_{VT2}\cdot f_{RVL}\cdot D_{VL}\cdot p_{F4}\right)\right)\right)s\cdot P_{V} \\ +\eta\cdot D_{HT1}\cdot f_{VT12}\cdot f_{RVL}\cdot D_{VL}\cdot p_{F4}\cdot s\cdot P_{V} \end{matrix}$$

$$R_{IHT2\_2}=f_{HP}\cdot f_{HD}\cdot f_{H\_S}\cdot f_{HT1}\left(D_{HT2}\cdot p_{F3}+\eta\cdot D_{HT2}\left(D_{VT2}\cdot p_{F3}+f_{VT2}\cdot f_{RVL}\cdot D_{VL}\cdot p_{F4}\right)\right)s\cdot P_{V}$$

$$\begin{matrix} R_{IHL\_2}=f_{HP}\cdot f_{HD}\cdot f_{H\_S}\cdot f_{HT1}\cdot f_{HT2}\left(f_{RHL}\left(D_{HL}+\eta\cdot D_{HL}\cdot D_{VL}\right)+\eta\cdot D_{RHL}\cdot f_{RVL}\cdot D_{VL}\right)p_{F4}\cdot s\cdot P_{V} \\ +f_{HP}\cdot f_{HD2}\left(f_{RHL}\left(D_{HL}+\eta\cdot D_{HL}\cdot D_{VL}\right)+\eta\cdot D_{RHL}\cdot f_{RVL}\cdot D_{VL}\right)p_{F4}\cdot s\cdot P_{V} \\ +f_{HP}\cdot f_{HD}\cdot f_{H\_S}\cdot f_{HT12}\left(f_{RHL}\left(D_{HL}+\eta\cdot D_{HL}\cdot D_{VL}\right)+\eta\cdot D_{RHL}\cdot f_{RVL}\cdot D_{VL}\right)p_{F4}\cdot s\cdot P_{V} \end{matrix}$$

Element *c* in matrix *M* is given by

$$c=R_{IVP\_1}+R_{IVD\_1}+R_{IVS\_1}+R_{IVT1\_1}+R_{IVT2\_1}+R_{IVL\_1}$$

whereby

$$\begin{matrix} R_{IVP\_1}=D_{VP}\cdot p_{F1}\cdot s\cdot\left(1-P_{V}\right) \\ R_{IVD\_1}=f_{VP}\cdot D_{VD}\cdot p_{F2}\cdot s\cdot\left(1-P_{V}\right) \\ R_{IVS\_1}=f_{VP}\cdot f_{VD}\cdot D_{VS}\cdot p_{F3}\cdot s\cdot\left(1-P_{V}\right) \\ R_{IVT1\_1}=f_{VP}\cdot f_{VD}\cdot f_{VS}\cdot D_{VT1}\cdot p_{F3}\cdot s\cdot\left(1-P_{V}\right) \\ R_{IVT2\_1}=f_{VP}\cdot f_{VD}\cdot f_{VS}\cdot f_{VT1}\cdot D_{VT2}\cdot p_{F3}\cdot s\cdot\left(1-P_{V}\right) \\ R_{IVL\_1}=f_{VP}\left(\begin{matrix} f_{VD}\cdot f_{VS}\cdot f_{VT1}\cdot f_{VT2}\cdot f_{RVL}\cdot D_{VL}+f_{VD2}\cdot f_{RVL}\cdot D_{VL} \\ +f_{VD}\cdot f_{VS}\cdot f_{VT12}\cdot f_{RVL}\cdot D_{VL}+f_{VD2}\cdot f_{RVL}\cdot D_{VL} \end{matrix}\right)\cdot p_{F4}\cdot s\cdot\left(1-P_{V}\right) \end{matrix}$$

Element *d* in matrix *M* is given by

$$d=R_{IVP\_2}+R_{IVD\_2}+R_{IVS\_2}+R_{IVT1\_2}+R_{IVT2\_2}+R_{IVL\_2}$$

whereby

$$\begin{matrix} R_{IVP\_2}=D_{VP}\cdot p_{F1}\cdot s\cdot P_{V} \\ R_{IVD\_2}=f_{VP}\cdot D_{VD}\cdot p_{F2}\cdot s\cdot P_{V} \\ R_{IVS\_2}=f_{VP}\cdot f_{VD}\cdot D_{VS}\cdot p_{F3}\cdot s\cdot P_{V} \\ R_{IVT1\_2}=f_{VP}\cdot f_{VD}\cdot f_{VS}\cdot D_{VT1}\cdot p_{F3}\cdot s\cdot P_{V} \\ R_{IVT2\_2}=f_{VP}\cdot f_{VD}\cdot f_{VS}\cdot f_{VT1}\cdot D_{VT2}\cdot p_{F3}\cdot s\cdot P_{V} \\ R_{IVL\_2}=f_{VP}\left(\begin{matrix} f_{VD}\cdot f_{VS}\cdot f_{VT1}\cdot f_{VT2}\cdot f_{RVL}\cdot D_{VL}+f_{VD2}\cdot f_{RVL}\cdot D_{VL} \\ +f_{VD}\cdot f_{VS}\cdot f_{VT12}\cdot f_{RVL}\cdot D_{VL}+f_{VD2}\cdot f_{RVL}\cdot D_{VL} \end{matrix}\right)\cdot p_{F4}\cdot s\cdot P_{V} \end{matrix}$$

For each of the 4 elements of *M* the *R*'s denote the contribution of the 6 infectious compartments *I_HP_*, *I_HD_*, *I_HS_*, *I_HT1_*, *I_HT2_*, *I_HL_* (humans without HIV) and *I_VP_*, *I_VD_*, *I_VS_*, *I_VT1_*, *I_VT2_*, *I_HL_* (humans with HIV) to *R_0_*, the basic reproduction number. *R_0_* is the largest eigenvalue of the next generation matrix *M*, whereby eigenvalues *λ_1,2_* are given by

yielding $\lambda_{1}=3.94$ and $\lambda_{2}=0$.

Thus, given the parameter estimates of this model, *R_0_* of the VL model is 3.94.

## Supplement C: Sensitivity analysis

Figure S2

See Figure S2 in high resolution as separate graphics.

**References**

48. Diekmann O, Heesterbeek JAP (2000) Mathematical epidemiology of infectious diseases: model building, analysis and interpretation. New York: Wiley.
